# A systematic review of service models and evidence relating to the clinically operated community-based residential mental health rehabilitation for adults with severe and persisting mental illness in Australia

**DOI:** 10.1186/s12888-019-2019-5

**Published:** 2019-02-04

**Authors:** Stephen Parker, Gordon Hopkins, Dan Siskind, Meredith Harris, Gemma McKeon, Frances Dark, Harvey Whiteford

**Affiliations:** 1 Metro South Addiction and Mental Health Services, Brisbane, QLD 4102 Australia; 2 School of Public Health, The University of Queensland, Herston, 4006 Australia; 3 School of Medicine, The University of Queensland, Herston, Australia

## Abstract

**Background:**

Clinically operated community-based residential rehabilitation units (Community Rehabilitation Units) are resource intensive services supporting a small proportion of the people with severe and persisting mental illness who experience difficulties living in the community. Most consumers who engage with these services will be diagnosed with schizophrenia or a related disorder. This review seeks to: generate a typology of service models, describe the characteristics of the consumers accessing these services, and synthesise available evidence about consumers’ service experiences and outcomes.

**Method:**

A systematic review was undertaken to identify studies describing Community Rehabilitation Units in Australia, consumer characteristics, and evidence about consumer experiences and outcomes. Search strings were applied to multiple databases; additional records were identified through snowballing. Records presenting unique empirical research were subject to quality appraisal.

**Results:**

The typology defined two service types, Community-Based Residential Care (C-BRC), which emerged in the context of de-institutionalisation, and the more recent Transitional Residential Rehabilitation (TRR) approach. Key differentiating features were the focus on transitional care and ‘recovery’ under TRR. Schizophrenia spectrum disorders were the most common primary diagnosis under both service types. TRR consumers were more likely to be male, referred from community settings, and less likely to be subject to involuntary treatment. Regarding outcomes, the limited quantitative evidence (4 records, 2 poor quality) indicated C-BRC was successful in supporting the majority of consumers transferred from long-term inpatient care to remain out of hospital. All qualitative research conducted in C-BRC settings was assessed to be of poor quality (3 records). No methodologically sound quantitative evidence on the outcomes of TRR was identified. Qualitative research undertaken in these settings was of mixed quality (9 records), and the four records exploring consumer perspectives identified them as valuing the service provided.

**Conclusions:**

While there is qualitative evidence to suggest consumers value the support provided by Community Rehabilitation Units, there is an absence of methodologically sound quantitative research about the consumer outcomes achieved by these services. Given the ongoing and increasing investment in these facilities within the Australian context, there is an urgent need for high-quality research examining their efficiency and effectiveness.

**Trial registration:**

PROSPERO (CRD42018097326).

**Electronic supplementary material:**

The online version of this article (10.1186/s12888-019-2019-5) contains supplementary material, which is available to authorized users.

## Introduction

The provision of clinically-operated rehabilitation in a community residential setting (Community Rehabilitation Units) reflects one approach to supporting people with severe and persisting mental illness to manage in the community. Most of the consumers who engage with mental health rehabilitation services will be diagnosed with schizophrenia or a related psychotic disorder [1, 2]. Schizophrenia is a low prevalence disorder associated with high levels of disability and societal costs [3, 4]. People affected by schizophrenia have varied responses to routine care, and many will continue to experience considerable functional deficits despite receiving optimal treatment. The positive symptoms, negative symptoms, and cognitive impairments associated with the disorder can detrimentally affect the capacity to maintain stable accommodation in the community.

Community Rehabilitation Units emerged in the context of de-institutionalisation. The initial goal was to assist long-stay inpatients in returning to community living in an appropriately supported ‘home-like’ environment [5]. Community Rehabilitation Units are now typically transitional in focus, aiming to help consumers to reside in more independent living situations by the time of discharge. Alternative approaches such as Housing First, which are focussed around the issue of homelessness rather than clinical rehabilitation, have been increasingly championed in North America. These models emphasise provision of permanent accommodation and the mobilisation of relevant support around a consumer’s own residence in the community [6–8]. Advocates for Housing First style approaches criticise clinically operated residential services for their limited evidence base [6, 9, 10] and ethical issues associated with making the provision of accommodation conditional on engagement with treatment [6, 7, 11]. Despite these criticisms, continued growth in the availability of different models of Community Rehabilitation Units such as Community Care Units and Community Rehabilitation Centres has occurred in Australia over the past two decades [1, 10, 12]. This discrepancy further highlights the need for increased definitional clarity and evidence regarding the characteristics and outcomes of consumers of these services [9].

This review aims to provide definitional clarity about the types of clinically operated Community Rehabilitation Units in the Australian context, and to examine the associated evidence base critically. Specifically, this review seeks to: (1) generate a typology of service models, (2) describe the characteristics of the consumers who access these services, and (3) synthesise the available evidence about consumers’ service experiences and outcomes.

## Methods

The systematic review followed the PRISMA guidelines [13]. The protocol for the review was registered with PROSPERO (CRD42018097326) [14].

### Eligibility criteria

Records were sought that described: (1) Australian Community Rehabilitation Units for people affected by severe and persisting mental illness, (2) the characteristics of the consumers engaging with these services, and (3) their service experiences and outcomes. Eligibility criteria for inclusion in the systematic review were a service focus on adults (18–65 years) with schizophrenia and related disorders. Rehabilitation units targeting consumers aged < 18 years or > 65 years, non-psychotic disorders (e.g. drug and alcohol, acquired brain injury, or physical rehabilitation) or specifically dual diagnosis consumers were excluded. These exclusion criteria related to the focus of the service model. There was no exclusion of consumer data from included records based on diagnosis or co-morbidity.

Databases were searched from 1995 onwards. This limit was applied as these services emerged in the context of the deinstitutionalisation process [15] with the first Community Care Unit opening in Victoria in 1996 [16]. No language specifiers were used. No exclusions were made based on study type, with emphasis placed instead on the assessment of the quality of included studies.

### Information sources

Parallel strategies were employed to identify relevant grey-literature using internet-based searches and published literature in academic databases (PubMed, CINAHL, PsycINFO and EmBASE).

### Search strategy

Full details of the search strategy are provided in Additional file 1: Literature search strategy. The initial search string applied to the PubMED database is illustrative of the process undertaken: “schizophrenia” [MeSH Terms] OR “schizophrenia” [All Fields]) AND (“rehabilitation” [Subheading] OR “rehabilitation” [All Fields] OR “rehabilitation” [MeSH Terms]) AND residential [All Fields]) AND (“1995/01/01” [PDAT]: “3000/12/31” [PDAT]).

### Study selection

Final extraction of records from all databases occurred on the 08/02/2018. Records were sequentially imported into an Endnote X8 database, and duplicates were removed. Two authors (SP & GH) independently screened records for eligibility at the title and abstract level. Additional records were identified through snowballing [17], with the reference lists of records assessed at the full-text level inspected to identify relevant documents. Attempts were made to contact authors of included records to identify relevant research and documentation.

### Quality appraisal

Records presenting unique quantitative and/or qualitative research data were subject to quality appraisal (full appraisals are provided in Additional file 2: Quality appraisal). Given the anticipated prominence of non-comparative methodologies in the published literature, the decision was made to rely on methodologically targeted quality appraisal instruments. Relevant checklists from the National Heart, Lung and Blood Institute (NIH) were used in the appraisal of ‘observational cohort and cross-sectional studies’, ‘systematic review and meta-analyses’, ‘case series studies’, ‘before-after (pre-post) studies with no control groups’, and ‘observational cohort and cross-sectional studies’ [18]. Studies presenting qualitative data, and the qualitative components of mixed-methods studies were assessed using the CASP Qualitative Checklist [19]. As the CASP Qualitative Checklist does not produce a global quality rating, the tripartite global rating system (Good, Fair, Poor) used in the NIH checklists was also applied to these records. Two of the authors (SP & GH), completed the quality assessments independently, and a final rating was determined following discussion to reach consensus. For studies where SP was one of the authors, DS and GH completed the quality appraisal.

### Data extraction and synthesis

Data extraction was completed independently by two authors (SP & GH). Discrepancies were resolved through discussion to reach consensus. Relevant content was extracted into matrices. Data of interest included descriptive information outlining service models (e.g. physical environment, philosophy of care, and ‘treatment and support’), service user characteristics (e.g. age, sex, diagnostic information, and chlorpromazine dose equivalence), and quantitative and qualitative research findings (e.g. outcomes, comparison with other services, consumer and staff perspectives). Descriptive data about service models were synthesised into a domains-based classification system [20], with the emergent typology derived from key emphases in the descriptive content. The typology was then used to organise the results into meaningful groups for interpretation.

Descriptive data regarding service user characteristics was pooled across studies, with means for service types and the associated models subsequently derived. Where data was available across both service types, differences in frequency of dichotomous variables was statistically compared using Chi-square or Fisher’s exact test. Fisher’s exact test was applied when the total sample size for pooled data was < 1000 cases [21] or if the frequency in any of the 2 × 2 cells was less than five [22]. A *p* ≤ .01 significance level was adopted to accommodate for the multiple comparisons and reduce the risk of Type 1 error. The absence of consistent documentation of standard deviations/standard errors for continuous variables (age and chlorpromazine dose equivalence) prohibited statistical comparison of the pooled means for these variables.

In relation to outcome data, no statistical synthesis was pre-specified or completed for the quantitative data. Findings from quantitative and qualitative studies were synthesised and tabulated.

## Results

**Fig. 1:**
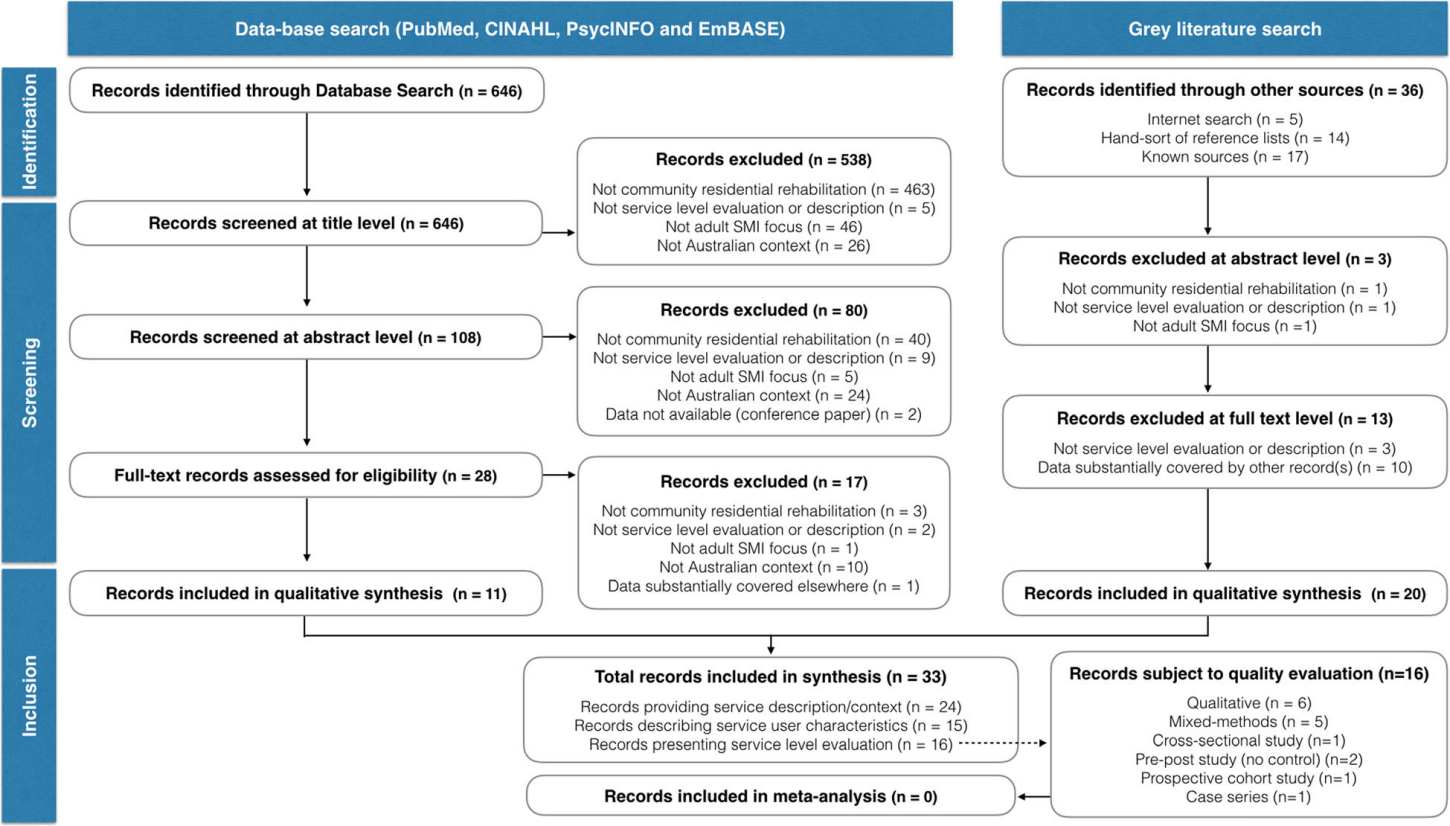
Flow diagram of data-extraction

As shown in Fig. 1, 33 records were included in the qualitative synthesis – 24 provided information about service description, 16 provided descriptive data on consumers, and 16 presented data on service experience and outcomes.

### Typology of Australian community rehabilitation units

From the 24 records providing service descriptions, two types of Community Rehabilitation Units were identified– the original Community-Based Residential Care (C-BRC) type operating from the mid-1990s until the early 2000s, and the more recent Transitional Residential Rehabilitation (TRR) type (see Table 1 & Additional file 3: Service types and models). These types and the associated named service models differed in the physical environment, philosophy of care, and the available treatment/support.

**Table 1:** Typology of key emphases of Australian Community Rehabilitation Units

Service Type	Service name	Timeframe	Location	Staffing				Built environment						Tenure		Philosophy				Treatment			
				Clinical		Integrated peer-support	NGO Partnership	Cluster housing	Apartment block	Repurposed community hospital	Single-occupancy	Dual-occupancy	Congregate	Permanent	Transitional	Rehabilitation	Support	Community access/partnership	Recovery	24-h staffing	Living skills	Individual therapies	Group therapies/programs
				Nursing	MDT																		
Community-based residential care	Community Residences^a^	1994-early 2000s	NSW	–	✓	–	–	✓	✓	–	✓	–	✓	✓	–	✓	✓	✓	–	✓	✓	–	–
	Community Care Unit^b^	1996-early 2000s	VIC	✓	✓	–	–	✓	–	–	–	–	–	✓	–	✓	✓	✓	–	✓	✓	✓	–
Transitional residential rehabilitation	Community Care Unit (CCU)^c^	Early 2000s+	VIC, QLD	–	✓	–	–	✓	–	–	✓	✓	–	–	✓	✓	✓	✓	✓	✓	✓	✓	✓
	Hawthorne House^d^	2006–2009	WA	–	✓	–	–	–	–	✓	–	–	✓	–	✓	✓	✓	✓	✓	✓	–	✓	–
	Community Rehabilitation Centre^e^	2007+	SA	–	✓	–	–	✓	–	–	✓	✓	✓	–	✓	✓	–	✓	✓	✓	✓	✓	–
	CCU (Integrated)^f^	2014+	QLD	–	✓	✓	–	✓	–	–	✓	✓	–	–	✓	✓	✓	✓	✓	✓	✓	✓	✓
	Community Recovery Program^g^	2014+	VIC	–	–	–	✓	✓	–	–	✓	–	–	–	✓	✓	✓	✓	✓	✓	✓	–	✓

^a^[23, 42, 43],

^b^[15, 24–28, 44],

^c^[1, 16, 24, 30, 39, 40, 45],

^d^[33],

^e^[31, 46, 47],

^f^[10, 29, 48],

^g^[32]

C-BRC service models emerged to meet the needs of institutionalised people with severe mental illness, predominantly schizophrenia, transitioning to community-based care. Service models associated with the C-BRC type were the Community Residences in New South Wales [23] and Community Care Units in Victoria [15, 24, 25]. These services focused on the provision of accommodation in a cluster housing configuration, in addition to rehabilitation and support to people affected by severe and persisting mental illness. Key features were the availability of 24-h clinical support and an individualised treatment focus on living skills development and community integration. Group-based therapies/programmes were explicitly de-emphasised under the original Community Care Unit model [26] and were not an essential feature of Community Residences. While C-BRC services were initially intended to provide a permanent residence, by the start of the twenty-first century their focus shifted towards a more transitional model of support [24, 27, 28].

The TRR type is distinct from C-BRC in its emphasis on the transitional nature of rehabilitation support as well as the inclusion of ‘recovery’ as part of the philosophy of care. The shift to a transitional focus coincided with: the initial cohort consumers in C-BRC services not requiring 24-h support on an ongoing basis [23, 24, 27]; most of the initial C-BRC-Community Care Unit cohort transitioning to more independent settings in the community [24, 27]; and an emergent emphasis on recovery-oriented care in mental health policy and planning frameworks [10, 29, 30]. Service models aligning with the TRR type are the Community Care Units in Queensland and Victoria [10], Community Rehabilitation Centres in South Australia [31], the Community Recovery Program in Victoria [32], and Hawthorne House in Western Australia [33]. Cluster housing remains the most common environmental configuration for TRR type services, and a focus on independent living or dual occupancy units predominates. Under TRR, there is greater emphasis on the availability of individual and group therapies/programs. Since 2014, deviations from the traditional clinical staffing configuration have emerged, including the substantial integration of peer support workers under the ‘Integrated Staffing Model’ and partnerships with Non-Government Organisations [32]. These alternative staffing configurations have been linked explicitly to the goal of further realising recovery-oriented care [29, 32, 34].

### Characteristics of consumers of community rehabilitation units in Australia

Data describing consumer characteristics from 15 records were organised according to the service typology in Tables 2 and 3 (additional detail available in Additional file 4: Consumer characteristics). There were limitations in the consistency and comparability of reported data. Variables reported in 50% or more of records for both C-BRC and TRR services were: age, sex, mode of referral, the presence of an involuntary treatment order, primary diagnosis, and total chlorpromazine dose equivalence of antipsychotic medication. Across both service types, most consumers were males aged in their 30s–40s with a primary diagnosis of a schizophrenia spectrum disorder.

**Table 2:** Community-based residential rehabilitation consumer characteristics: Demographics, restrictive practice and referrals

Type	Service name	Timeframe	Age			Sex			Origin			ATSI^d^			Employment			MHA^e^			Guardian			Referral		
			Studies (n/N)^c^	Pooled sample size (n)	Average (years)	Studies (n/N)	Sample size (n)	Male (%)	Studies (n/N)	Pooled sample size (n)	Australian Born (%)	Studies (n/N)	Pooled sample size (n)	Proportion with ATSI identification	Studies (n/N)	Pooled sample size (n)	Unemployed (%)	Studies (n/N)	Pooled sample size (n)	Involuntary Treatment Order (%)	Studies (n/N)	Pooled sample size (n)	Guardianship order (%)	Studies (n/N)	Pooled sample size (n)	Non-inpatient/community-based (%)
C-BRC^a^	Community Residences	1994-early 2000s	1/1	47	41	1/1	47	53%	1/1	47	83%	0/1	–	–	0/1	–	–	0/1	–	–	0/0	–	–	1/1	47	0%
	Community Care Unit (CCU)	1996-early 2000s	4/4	361	40	4/4	361	62%	0/4	–	–	0/4	–	–	0/4	–	–	3/4	230	86%	0/4	–	–	4/4	361	0%
	Total	1994-early 2000s	5/5	408	40	5/5	408	61%	1/5	47	83%	0/5	–	–	0/5	–	–	3/5	230	86%	0/5	–	–	5/5	408	0%
TRR^b^	CCU^f^	Early 2000s-2013	5/5	453	37	4/5	338	73%	3/5	262	87%	4/5	338	11%	0/5	–	–	5/5	453	61%	4/5	377	46%	0/5	–	–
	Hawthorne House	2006–2008	1/1	39	33	1/1	39	49%	0/1	–	–	0/1	–	–	0/1	–	–	0/1	–	–	0/1	–	–	1/1	39	0%
	Community Rehabilitation Centre^g^	2007+	1/1	238	32	1/1	238	75%	0/1	–	–	1/1	238	5%	0/1	–	–	1/1	238	7%	0/1	–	–	1/1	266	81%
	CCU +/− Integrated Staffing Model^h,i^	2014+	3/3	420	36	3/3	420	73%	2/3	396	84%	2/3	396	10%	2/3	265	73%	3/3	420	60%	2/4	396	37%	3/3	420	47%
	Total	Early 2000s+	10/10	1150	35	9/10	1035	73%	5/10	658	85%	7/10	972	9%	2/10	265	73%	9/10	1111	49%	6/10	773	41%	6/10	725	57%

^a^*C-BRC *Community-Based Residential Care,

^b^*TRR* Transitional Residential Rehabilitation, Nil consumer data is available for the TRR Community Recovery Program model,

^c^Relevant studies: C-BRC Community Residences [23], C-BRC CCU [15, 25–27], TRR CCU [1, 49–53], TRR Hawthorn House [33], TRR Community Rehabilitation Centre [31], TRR CCU +/− Integrated Staffing Model [40, 54, 55],

^d^*ATSI* persons identifying as being of Aboriginal and/or Torres Strait Islander descent,

^e^*MHA* Mental Health Act status,

^f^At least partially overlapping data from included records is anticipated,

^g^Sample size varies with data availability, for the single available record, and ‘Referral’ considers all consumers referred during the study period and not only those accepted into care,

^h^Pooling across sites operating and not operating an Integrated Staffing Model was necessitated by how the data was presented in the included records,

^i^At least partially overlapping data from included records is anticipated due to the inclusion of cross-sectional data of current service users across multiple time points

**Table 3:** Community-based residential rehabilitation consumer characteristics: Diagnosis, comorbidity, symptoms, functioning and medication

Type	Service name	Timeframe	Primary Diagnosis			Comorbidity												Symptoms and functioning						Medication		
						Substance use^e^			Developmental			Personality			Physical illness			LSP-16^j^			HoNOS^k^					
			Studies reporting (n/N)^c^	Pooled sample size (n)	Schizophrenia-spectrum^d^	Studies reporting (n/N)	Pooled sample size (n)	Co-morbid substance use	Studies reporting (n/N)	Pooled sample size (n)	Developmental disorder	Studies reporting (n/N)	Pooled sample size (n)	Personality disorder	Studies reporting (n/N)	Pooled sample size (n)	Significant physical illness	Studies reporting (n/N)	Pooled sample size (n)	LSP-16 (Total)	Studies reporting (n/N)	Pooled sample size (n)	HoNOS (Total)	Studies reporting (n/N)	Pooled sample size (n)	Chlorpromazine dose eq. (mg)
C-BRC^a^	Community Residences	1994-early 2000s	1/1	47	98%	0/1	–	–	1/1	47	2%	0/1	–	–	0/1	–	–	0/1	–	–	0/1	–	–	1/1	47	1127
	Community Care Unit (CCU)	1996-early 2000s	3/4	230	95%	1/4	20	15%	1/4	20	15%	0/4	–	–	1/4	20	20%	0/4	–	–	0/4	–	–	2/4	145	797
	Total	1994-early 2000s	4/5	277	96%	1/5	20	15%	2/5	47	8%	0/5	–	–	1/5	20	20%	0/5	–	–	0/5	–	–	3/5	192	878
TRR^b^	CCU^e^	Early 2000s-2013	5/5	453	90%	3/5	262	26%	0/5	–	–	0/5	–	–	5/5	453	41%	1/5	115	21.0	1/6	115	12.7	3/6	285	647
	Hawthorne House	2006–2008	1/1	39	71%	0/1	–	–	0/1	–	–	0/1	–	–	0/1	–	–	0/1	–	–	0/1	–	–	0/1	–	–
	Community Rehabilitation Centre^g^	2007+	1/1	266	80%	1/1	230	4%	1/1	230	2%	1/1	230	4%	1/1	230	3%	0/1	–	–	1/1	126	16.5	0/1	–	–
	CCU +/− Integrated Staffing^h^	2014+	3/3	420	87%	3/3	420	28%	2/3	179	5%	2/3	179	6%	2/3	396	52%	1/3	24	13.0	1/3	24	12	3/3	420	591
	Total	Early 2000s+	10/10	1178	86%	9/10	912	21%	3/10	409	3%	3/10	409	5%	9/10	1079	37%	2/10	139	19.6	4/10	265	14.4	6/11	705	614

^a^*C-BRC* Community-Based Residential Care,

^b^*TRR* Transitional Residential Rehabilitation, nil consumer data is available for the TRR Community Recovery Program model,

^c^Relevant studies: C-BRC Community Residences [23], C-BRC CCU [15, 25–27], TRR CCU [1, 49–53], TRR Hawthorn House [33], TRR Community Rehabilitation Centre [31], TRR CCU +/− Integrated Staffing Model [40, 54, 55],

^d^Equivalent documented diagnoses to the ICD-9 F20–29.x classifications,

^e^Documented substance use disorder excluding tobacco-related disorders,

^f^At least partial overlap of data from contributing records is anticipated, ^g^Sample size varies with data available for the single included record,

^h^Pooling across sites operating and not operating an Integrated Staffing Model was necessitated by how the data was presented in the included records,

^i^At least partially overlapping data from included records is anticipated due to the inclusion of cross-sectional data of current service users across multiple time points,

^j^*LSP-16* Life Skills Profile 16 [56],

^k^*HoNOS* Health of the Nation Outcome Scale [57]

Differences arose in the characteristics of consumers from the C-BRC and TRR service types. TRR consumers were significantly more likely to be male (*X*^2^ 19.98, *p* < .01) and to be referred from the community rather than an inpatient service (Fisher exact test, *p* < .01). Schizophrenia spectrum disorders were the most common primary diagnosis across both service types, although this was significantly less frequent within the TRR service type (*X*^2^ 21.24, *p* < .01). TRR consumers were also significantly less likely to be subject to an involuntary treatment order (*X*^2^ 106.24, *p* < .01). C-BRC and TRR consumers did not differ significantly with respect to the frequency of being born in Australia (Fisher exact test, *p* = 0.68), or rates of comorbid disorders (substance use (Fisher exact test *p* = 0.78), developmental disorders (Fisher exact test, *p* = 0.07), and physical illness (Fisher exact test, *p* = 0.16).

Statistical comparison of the mean values for age and chlorpromazine dose equivalence between the C-BRC and TRR groups could not be performed as standard deviations/standard errors were not reliably reported. Descriptive statistics suggest divergence between the C-BRC and TRR services, in that consumers entering the more recent TRR services were younger (pooled x̅ 35, range of available means 30–39; versus 40 years, range of available means 38–43), and on lower total chlorpromazine dose equivalence of antipsychotic medication (pooled x̅ 614, range of available means 573-728 mg/day; versus 878 mg/day, range of available means 936-1127 mg/day).

### Service experiences and outcomes

With regards to quantitative research, only six records were identified. Findings from these studies are detailed in Table 4 (with additional detail in Additional file 5: Included research). Meta-analysis and statistical assessment of publication bias and sensitivity analysis were not appropriate given the insufficient number of comparable studies [35]. Follow-up outcome data was available from two C-BRC studies assessed as ‘fair’ quality. These studies found most consumers remained in the community at long-term follow-up [23, 24], with one study noting high levels of ongoing disability [24]. One ‘poor’ quality report presented follow-up outcome data for the TRR type; the favourable pre-post outcomes with respect to reduced inpatient bed-days and symptoms observed in this study should be interpreted cautiously [31].

**Table 4:** Quantitative research findings relating to consumer outcomes and experiences of community-based residential rehabilitation

Service type	Service name	Research focus	Studies reporting					Finding(s)
			(n/N)^c^	Quality			Source(s)	
				Good	Fair	Poor		
Community-Based Residential Care	CCU^a^	Symptom stability (initial)	2/3	1	–	1	[15, 26]	▪ No significant change observed in resident symptoms or functioning over the initial 12-months, or for a subsample of initially transferred residents over a subsequent 12-month period.
		Quality of life	1/3	–	–	1	[15]	▪ Significant improvement at 1-year post-transition from long-stay inpatient care.
		Follow-up outcomes	1/3	–	1	–	[24]	▪ High levels of ongoing disability and dependence on clinical services 8-years following service entry.
	Community Residences	Follow-up outcomes	1/1	–	1	–	[23]	▪ 18% of residents required admission to inpatient psychiatric care within 2-years of transfer, and an additional 28% required admission to inpatient psychiatric care in the subsequent 4-years. ▪ 6-years post-transition 85% of residents continued to reside in the community, and none of these people had an ongoing requirement for 24-h supervision. Additionally, significant improvements in quality of life, and reductions in medication usage were noted for community-based residents.
Transitional Residential Rehabilitation	CCU	Comparison to inpatient rehabilitation	1/1	–	1	–	[1]	▪ Compared to consumers engaged in inpatient rehabilitation CCU consumers were significantly: younger; less likely to be subject to involuntary treatment and guardianship orders; less likely to be classified as being of a moderate-to-high risk of violence; lower on levels of symptoms (HoNOS) and disability (LSP-16).
	CRC^b^	Follow-up outcomes	1/1	–	–	1	[31]	▪ Significant reductions in inpatient bed-days, symptoms and functioning (HoNOS, all subscales except behaviour) when comparing the 6-month period pre- and post-CRC care.

^a^Community Care Unit (CCU)

^b^Community Rehabilitation Centre (CRC)

^c^The denominator is the number of studies undertaken under the specified Service Name; the numerator is the subset of studies undertaken with the relevant Research Focus

Twelve records presented qualitative research findings that considered a range of stakeholder perspectives (consumer, staff and family). Findings are detailed in Table 5 (see also Additional file 5: Included research), and the findings relating to consumer experiences, which reflect the majority focus of the qualitative research, is additionally summarised in text. Consumer reflections about their expectations and experience of care at Community Rehabilitation Units were consistently positive, regardless of service type and name. Two studies explored long-term follow-up in C-BRC services. These studies described ongoing challenges faced by consumers, including impoverished social networks [23, 24] and accommodation instability [24]. All records presenting qualitative findings relating to C-BRC services were assessed to be of poor quality. Seven of the nine records considering TRR models focused on Community Care Units, which were of mixed quality (Good = 4, Fair = 1, Poor = 2); the remaining two records from non-Community Care Unit settings were assessed to be of poor quality.

**Table 5:** Qualitative research findings relating to consumer outcomes and experiences of service

Service type	Service name	Research focus	Studies reporting					Finding(s)
			(n/N)^d^	Quality			Source(s)	
				Good	Fair	Poor		
Community-Based Residential Care	CCU^a^	Consumer perspective	1/2	–	–	1	[24]^b^	▪ 8-year follow-up post service entry identified themes of disempowerment, instability in accommodation and social networks, issues with continuity of care, and loss were identified.
	Community Residences		1/2	–	–	1	[23]^b^	▪ 6-year follow-up found residents describing increased freedom, but also difficulties enhancing social networks, absence of new goals and lack of expectation of change in life circumstances.
			1/1	–	–	1	[42]	▪ Residents express preference for community living to long-term inpatient care in the initial period following transfer.
		Staff perspective	1/1	–	–	1	[42]	▪ Staff identify the process of new skill acquisition for formally de-institutionalised residents as ‘not easy’ and acknowledged slow but continual progress, as well as the reducing support needs for residents over time.
Transitional Residential Rehabilitation	CCU	Consumer perspective	4/4	2	–	2	[25, 39, 40, 58]	▪ The services are viewed favourably by consumers entering and engaging with them, particularly in comparison to inpatient psychiatric care. Positive aspects of the care environment include increased opportunity for independence and activity engagement and availability of caring staff.
			2/4	1	–	1	[40, 58]	▪ Consumers understand the transitional and rehabilitation foci of the service. Additionally, they view it as providing an environment facilitating social interaction, friendship and mutual support between co-residents.
			1/4	1	–	–	[40]	▪ Content analysis found that most consumers had been involved in the decision to come to the CCU, and the most common reason for engagement was accommodation instability rather than the opportunity to engage in rehabilitation.
			1/4	1	–	–	[39]	▪ Favourable expectations of the increased availability of Peer Support Workers at the study sites trialling an Integrated Staffing Model.
		Staff perspective	3/3	2	1	–	[30, 45, 48]	▪ Staff understandings of these services are consistent with the designated service models.
			1/3	–	1	–	[30]^c^	▪ Content domains of the recovery concept identified as: a shared vision of recovery as ‘a continuous journey’; the importance of clinicians ‘promoting hope’, shifting emphasis from rehabilitation to ‘promoting autonomy and self-determination’, the centrality of ‘meaningful engagement and collaborative partnerships’, ‘holistic and personalised care’, and ‘community participation and citizenship’.
			1/3	1	–	–	[45]	▪ Four themes relating to the staff concept of the CCU were identified: ‘rehabilitation is different to treatment’, a ‘positive transitional space’, ‘they (consumers) have to be ready to engage’, and ‘recovery is central to rehabilitation practice’. ▪ Burnout and external pressure from the broader mental health system limit the ability to deliver recovery-oriented rehabilitation.
			1/3	1	–	–	[48]	▪ Commencing staff have positive expectations of the integration of peer support with clinical staff under the Integrated Staffing Model; anticipating the CCU to be ‘a place of mutual learning and co-development’, ‘a temporary and transitional place’, and provide a simulacra of community living.
		Family perspective	1/1	–	–	1	[58]	▪ Service viewed favourably in the single family member perspective presented.
	CRC^a^	Multiple stakeholder perspectives	1/1	–	–	1	[31]	▪ Consumers understand the transitional and rehabilitation foci of the service. ▪ Staff understanding of the service is consistent with the designated service models.
	HH^a^	Multiple stakeholder perspectives	1/1	–	–	1	[33]	▪ The service was viewed favourably by consumers and their families.

^a^Community Care Unit (CCU), Community Rehabilitation Centre (CRC), Hawthorn House (HH).

^b^Data from mixed methods study with a primary quantitative emphasis.

^c^Exploratory study with staff providing the majority stakeholder perspective.

^d^The denominator is the number of studies undertaken under the specified Service Name; the numerator is the subset of studies undertaken with the relevant Research Focus

## Discussion

This systematic review identified core features of Australian Community Rehabilitation Units as the provision of 24-h rehabilitation focused support in a community-based residential setting to mental health consumers primarily diagnosed with a schizophrenia spectrum disorder. Two distinct service types were identified: the original C-BRC type that emerged in the context of deinstitutionalisation, and the more recent TRR type which replaced this from the early 2000s. TRR services differed from C-BRC in their transitional (time-limited) focus and emphasis on recovery-oriented care. Significant differences were present in the profile of consumers engaged with TRR and the earlier C-BRC services. Specifically, TRR consumers are more likely to be male, referred from the community and treated voluntarily, and less likely to have a primary diagnosis of a schizophrenia spectrum disorder. The extent to which these changes relate to shifts in relevant mental health policy needs to be considered.

Quantitative research exploring the outcomes of Community Rehabilitation Units is limited. There is evidence to suggest that C-BRC services met their original goal of avoiding hospitalisation for most consumers initially transferred from long-stay inpatient care. Additionally, the need for 24-h supervision declined over time. The transition to community-based care was associated with improvements in quality of life. There is an absence of methodologically sound quantitative evidence considering the outcomes achieved for consumers engaged with the current TRR service type.

Methodological concerns in qualitative research conducted in these settings were commonly identified. All qualitative research conducted in C-BRC settings and in non-Community Care Unit TRR settings was assessed to be of ‘poor’ quality. Qualitative research consistently reported favourable expectations and experiences of care at Community Rehabilitation Units by consumers and their families across both service types. Consumers and staff of TRR services articulate understandings of the service consistent with the designated service models. However, there is an absence of follow-up qualitative data from former TRR consumers, and that available for the original C-BRC cohort suggests consumers may continue to struggle with issues such as social isolation, accommodation instability and disability following the receipt of intensive rehabilitation support.

### Putting the findings in context

The typology enhances understanding of the nature and function of Australian Community Rehabilitation Units for people affected by severe and persisting mental illness. Within the Australian context the typology identified potential bases of service non-comparability, whereby even identically named services were discrepant from each other. The matrix conceptually grouping the key emphases of services models supports the appropriateness of comparing extant Australian service models in future research. Additionally, differential features associated with sub-types and specific service models will facilitate the exploration of differences in service outcomes that may emerge. A relevant example of this may be the impact of novel staffing models being trialled, such as the integration of peer support work within the multidisciplinary team and non-government organisation partnerships.

The availability of domains-based classification presents an opportunity to synthesise existing research internationally, and for future research collaboration. By identifying the key emphases of Australian service models, the typology will allow identification of the extent to which these models are similar, and the appropriateness of comparing and synthesising current and future quantitative and qualitative research findings through meta-analytic methods. One potential opportunity for comparison is the overlap identified between the two Australian Community Rehabilitation Unit types, and two of the five types of supported accommodation emerging in the recent English classification system of supported accommodation - ‘The Simple Taxonomy for Supported Accommodation’ (STAX-SA) [36]. C-BRC aligns with ‘Type 1,’ which is characterised by ‘staff on site’, ‘high support’, ‘limited emphasis on move-on’ and ‘congregate setting’. TRR aligns with ‘Type 2,’ which is distinguished from ‘Type 1’ by its ‘strong emphasis on move on’. This alignment suggests the appropriateness of comparing Australian TRR services and English services classified as ‘Type 1’ in future research.

The limitations in research exploring the outcomes for TRR consumers are concerning given the increasing investment in these services [1, 10, 12] and ongoing adaptation of service models in Australia [34]. There continues to be a strong mental health policy emphasis on the provision of community-based alternatives to inpatient psychiatric services [37, 38]. The findings of this systematic review support concerns that this policy drive is uncoupled from the evidence base [9]. Multiple stakeholders and outcomes are relevant to understanding whether these services are achieving their purpose and offering value for money. Important outcome considerations relating to consumers include functional recovery (e.g. gains in employment, accommodation stability, and living skills), clinical recovery (e.g. improvement on symptomatic measures), as well as consumer defined recovery and quality of life. The outcome of service provision on carer burden is another relevant consideration. Outcomes of relevance to service planners include reductions in inpatient psychiatric bed days, emergency department presentations, and other indexes of service utilisation. None of these outcomes are adequately addressed in the literature.

The consistent finding of positive expectations and reflections on the experience of Community Rehabilitation Units by consumers across qualitative studies provides some support for the value and acceptability of these services. Additionally, available research exploring consumer’s commencement expectations of TRR services found they understood the model [39, 40], and most were actively involved in the decision to come [40]. These findings counter the international trend toward de-emphasising and de-valuing clinically focused TRR in favour of an emphasis on the provision of permanent housing and the mobilisation of relevant support around the consumer [6–8]. However, the lack of follow-up qualitative and quantitative research examining the associated short- and long-term outcomes for Community Rehabilitation Units remains problematic. There are significant risks associated with increased investment and adaptation of service models that remain largely untested.

### Limitations

This review deliberately focused on Community Rehabilitation Units for people affected by severe and persisting mental illness in the Australian context. The review did not consider other types of accommodation and support that may be provided to this group, including substance use rehabilitation, non-clinical psychosocial rehabilitation services delivered by non-government organisations, and non-rehabilitation focussed supported accommodation. While these alternative residential services are outside the scope of the present review, a comprehensive understanding of the service array is critical for answering the complex questions of what works for whom, under what circumstances, and why.

The two defined service types (C-BRC and TRR) reflect changes in the approach to these services over time, rather than two distinct types that are currently operating. This limits the utility of the C-BRC type to efforts directed towards understanding historical aspects of care.

While the process of pooling service user data facilitated statistical comparison between the C-BRC and TRR types, there are several limitations associated with this approach. At least partial overlap of consumer characteristic data is anticipated due to the combination of cross-sectional and service commencement data covering overlapping time periods. Additionally, the use of cross sectional data may create bias towards more severely impaired consumers who received longer durations of residential care.

Significant gaps and methodological limitations in the literature were identified, particularly with regards to qualitative research for C-BRC services and quantitative research for TRR services. There were no randomised controlled trials, and naturalistic observational designs predominated. Given the socially complex nature of these services the absence of studies involving randomisation, blinding and controls was anticipated. Moreover, there are ethical and practical barriers to attempting randomized controlled trials of such services [41].

## Conclusions

Community Rehabilitation Units in Australia fulfilled their original purpose of supporting de-institutionalised consumers to avoid hospitalisation. However, the focus of these units has shifted to the provision of transitional recovery-oriented care since the early 2000s. The profile of consumers currently accessing these services differs significantly from the original cohort. Although there is qualitative evidence to suggest that consumers value the support provided, there is no methodologically sound research quantifying the outcomes achieved. Given the ongoing and increasing investment in these services in the Australian context, there is an urgent need for high-quality research to examine the efficiency and effectiveness of these services. Such an evidence-base would be invaluable to informing policy decisions regarding the allocation of funding within the mental health service array.

## Additional files

Additional file 1: Literature Search Strategy. (PDF 403 kb)

Additional file 2: Quality appraisal. (PDF 156 kb)

Additional file 3: Service types and models. (PDF 99 kb)

Additional file 4: Consumer characteristics. (PDF 457 kb)

Additional file 5: Included research relating to community-based and clinically operated residential rehabilitation for people affected by schizophrenia and related disorders in Australia. (PDF 559 kb)

## Abbreviations

ATSI: Persons identifying as being aboriginal or torres strait islander
C-BRC: Community-based residential care
CCU: Community Care Unit
CR: Community residences
CRC: Community Rehabilitation Centre
HH: Hawthorn House
NIH: National Heart, Lung and Blood Institute
SPMI: Severe and persisting mental illness
STAX-SA: Simple taxonomy for supported accommodation
TRR: Transitional residential rehabilitation
