# Supplementary material for: A systematic review of service models and evidence relating to the clinically operated community-based residential mental health rehabilitation for adults with severe and persisting mental illness in Australia
Source: BMC Psychiatry. 2019 Feb 4;19:55. doi: 10.1186/s12888-019-2019-5 (PMC6360669; doi:10.1186/s12888-019-2019-5)
Supplement: Supplementary file 1 — Literature Search Strategy. (PDF 403 kb) [file 12888_2019_2019_MOESM1_ESM.pdf]

Additional file 1: Search strategy.

Grey-literature: Initial internet search strategy

Database	Date	Purpose	Terms	Output		
Google	16-10-15	Scoping – identification of relevant service types for subsequent search construction<br><br>AND<br><br>Identification of relevant grey-literature sources for inclusion.	rehabilitation AND residential AND community AND psychiatric OR psychosocial OR "mental health" (limited to the country Australia)	Limited to consideration of the first 100 records listed by relevance	Examples of out of scope records/services:<br>▪ Youth Residential Rehabilitation (Victoria) (e.g. https://www2.health.vic.gov.au/mental-health/mental-health-services/services-by-type/mental-health-community-support-services/youth-residential-rehabilitation-services)<br>▪ Peer Recovery Communities (non-clinical) and Prevention and Recovery Care PARC (clinical but step-up/step-down)<br>▪ Non-clinical residential rehabilitation programs, e.g. OTCP and Benambra Residential Service(NSW)<br>▪ Non-community based inpatient services (e.g. SMHRU, Secure Extended Care Units)<br>▪ Residential Rehabilitation & Recovery services Level 5 (Tasmania) which approximate PARC (i.e. step-up/step-down care)	https://www.mindaustralia.org.au/assets/docs/Resources/Mind%20Peer%20Recovery%20f_web.pdf<br>http://www.otcp.com.au/about-us/company-overview/<br>http://www.dhhs.tas.gov.au/__data/assets/pdf_file/0003/38982/RRRS_Model_of_Care_March_2008a.pdf
					Potentially in scope records/services identified:<br>▪ Community Mental Health Rehabilitation Service and Community Rehabilitation Centre (SA)<br>▪ Adult Residential Rehabilitation (Vic)<br>▪ Community Care Units and Residential rehabilitation services (Vic)<br>▪ Residential Recovery Program (Ballarat) – a Community Care Unit type service<br>▪ Brian Hennessy Rehabilitation Centre BHRC / Brian Hennessy House (ACT)<br>▪ Paterson, S (2002) A Review of Anglesey Interim Community Rehabilitation Facility. Preliminary report. Department of Health, Government of Western Australia, Perth.	▪ http://www.sahealth.sa.gov.au/wps/wcm/connect/34f13180442229d7aae7eeac7d3e3720/Community+Mental+Health+Rehabilitation+Service_Brochure.pdf?MOD=AJPERES&CACHEID=34f13180442229d7aae7eeac7d3e3720<br>▪ https://www2.health.vic.gov.au/getfile/?sc_itemid={2967ACFF-25A8-4369-A388-4087FAAD9B17}&title=Review%20of%20the%20PDRSS%20Day%20Program,%20Adult%20Residential%20Rehabilitation%20and%20Youth%20Residential%20Rehabilitation%20Services<br>▪ http://www.betterhealth.vic.gov.au/bhcv2/bhcarticles.nsf/pages/Mental_illness_services_residential_care<br>▪ https://www.bhs.org.au/node/266<br>▪ http://www.mhf.org.au/services-directory/item/brian-hennessy-rehabilitation-centre<br>▪ http://www.health.wa.gov.au/docreg/Education/Population/Health_Problems/Mental_Illness/A_recovery_vision_for_rehabilitation.pdf

Database search strategy:

Database	Final search date	Purpose	Search string(s)	Unique records identified
**PubMed**	08-02-18	Broad capture of relevant records	(("schizophrenia"[MeSH Terms] OR "schizophrenia"[All Fields]) AND ("rehabilitation"[Subheading] OR "rehabilitation"[All Fields] OR "rehabilitation"[MeSH Terms]) AND residential[All Fields]) AND ("1995/01/01"[PDAT] : "3000/12/31"[PDAT])	318
		Focussed capture using identified services from initial internet search	"community rehabilitation centre"[All Fields] OR “community mental health rehabilitation service” [All fields] OR "community care unit"[All Fields] OR "Residential recovery program"[All Fields] OR “hennessey house” [All Fields] OR “hennessey rehabilitation centre” [All Fields] OR “Anglesey interim rehabilitation” [All Fields] AND ("1995/01/01"[PDAT] : "3000/12/31"[PDAT])	
**CINAHL**	08-02-18	Broad capture of relevant records	rehabilitation and residential and schizophrenia and community (Published Date: 19950101-20151231)	65
		Focussed capture using identified services from initial internet search	"community rehabilitation centre" OR "community mental health rehabilitation service" OR "community care unit" OR "residential recovery program" OR "hennessey house" OR "hennessey rehabilitation centre" OR "anglesey interim rehabilitation" (Published Date: 19950101-20151231)	
**PsycINFO**	08-02-18	Broad capture of relevant records	Any Field: schizophrenia *AND* Any Field: residential *AND* Any Field: rehabilitation *AND* Year: 1995 *To* 9999	100
		Focussed capture using identified services from initial internet search	Any Field: "community rehabilitation centre" OR "community mental health rehabilitation service" OR "community care unit" OR "residential recovery program" OR "hennessey house" OR "hennessey rehabilitation centre" OR "anglesey interim rehabilitation" **Year**: 1995 *To* 9999	
**EmBASE**	08-02-18	Broad capture of relevant records	'schizophrenia'/exp OR schizophrenia AND residential AND ('rehabilitation'/exp OR rehabilitation) AND [1995-2015]/py	163
		Focussed capture using identified services from initial internet search	‘community rehabilitation centre’ OR ‘community mental health rehabilitation service’ OR ‘community care unit’ OR ‘residential recovery program’ OR ‘hennessey house’ OR ‘hennessey rehabilitation centre’ OR ‘anglesey interim rehabilitation’ AND [1995-2015]/py	
**TOTAL**				**646**
