# Supplementary material for: A systematic review of service models and evidence relating to the clinically operated community-based residential mental health rehabilitation for adults with severe and persisting mental illness in Australia
Source: BMC Psychiatry. 2019 Feb 4;19:55. doi: 10.1186/s12888-019-2019-5 (PMC6360669; doi:10.1186/s12888-019-2019-5)
Supplement: Supplementary file 2 — Quality appraisal. (PDF 156 kb) [file 12888_2019_2019_MOESM2_ESM.pdf]

Authors	Year	Type	Rating tool #1	Rater #1	Rater #2	Rater #3	Rating tool #2	T2 Rater #1	T2 Rater #2
Barnett et al	2011	Grey literature	NIHL Checklist - Quality Assessment Tool for Before-After (Pre-Post) Studies with no control group	Poor	Poor	N/A	CASP - Qualitative Checklist	Poor	Poor
Chopra et al	2011	Mixed methods (case series and qualitative)	NIHL Checklist - Quality Assessment Tool for Case Series Studies	Fair	Fair	N/A	CASP - Qualitative Checklist	Poor	Poor
Farhall et al	2003	Prospective cohort study	NIHL Checklist - Quality Assessment Tool for Observational Cohort and Cross-Sectional Studies	Good	Good	N/A	N/A	N/A	N/A
Hamden et al	2011	Pre-post studies with no control	NIHL Checklist - Quality Assessment Tool for Before-After (Pre-Post) Studies with no control group	Poor	Poor	N/A	N/A	N/A	N/A
Hobbs et al	2002	Mixed methods (prospective cohort and qualitative)	NIHL Checklist - Quality Assessment Tool for Observational Cohort and Cross-Sectional Studies	Fair	Fair	N/A	CASP - Qualitative Checklist	Poor	Poor
McKenna et al	2016	Qualitative study	CASP - Qualitative Checklist	Fair	Fair	N/A	N/A	N/A	N/A
Meehan et al	2016	Cross sectional study	NIHL Checklist - Quality Assessment Tool for Observational Cohort and Cross-Sectional Studies	Fair	Fair	N/A	N/A	N/A	N/A
Meurck et al	2018	Qualitative study	CASP - Qualitative Checklist	Fair	Fair	N/A	N/A	N/A	N/A
Munroe et al	2007	Systematic review	CASP - Qualitative Checklist	Poor	Poor	N/A	N/A	N/A	N/A
Newton	2000	Qualitative study	CASP - Qualitative Checklist	Poor	Poor	N/A	N/A	N/A	N/A
Parker et al	2018	Qualitative study	CASP - Qualitative Checklist	Good	Good	N/A	N/A	N/A	N/A
Parker et al	2017	Qualitative study	CASP - Qualitative Checklist	Good	Good	N/A	N/A	N/A	N/A
Parker et al	2016	Qualitative study	CASP - Qualitative Checklist	Good	Good	N/A	N/A	N/A	N/A
Smith	2009	Grey literature	NIHL Checklist - Quality Assessment Tool for Case Series Studies	Poor	Poor	N/A	CASP - Qualitative Checklist	Poor	Poor
Trauer et al	2001	Case series	NIHL Checklist - Quality Assessment Tool for Case Series Studies	Good	Good	N/A	N/A	N/A	N/A
Trauer et al	2001	Pre-post studies with no control	NIHL Checklist - Quality Assessment Tool for Before-After (Pre-Post) Studies with no control group	Poor	Poor	N/A	N/A	N/A	N/A

NIHL Checklist - Quality Assessment Tool for Before-After (Pre-Post) Studies with no control group

Study: **Barnett K, Guiver N, Cheok F. Evaluation of the Three Community Rehabilitation Centres: FINAL REPORT. In: Health S, editor. South Australia: SA Health; 2011.**

	RATER 1				RATER 2			
**Criteria**	**Yes**	**No**	**Other (CD, NR, NA)***	**Justification**	**Yes**	**No**	**Other (CD, NR, NA)***	**Justification**
1. Was the study question or objective clearly stated?	1	0		Detailed in terms of reference	1	0		Objective is stated in the terms of reference
2. Were eligibility/selection criteria for the study population prespecified and clearly described?	1	0		All consumers entering the service over a specified time period	1	0		All residents of the CRC's which were at 80-90% occupancy during the time period
3. Were the participants in the study representative of those who would be eligible for the test/service/intervention in the general or clinical population of interest?	1	0		All consumers entering the service over a specified time period	1	0		As above
4. Were all eligible participants that met the prespecified entry criteria enrolled?	1	0		All consumers entering the service over a specified time	1	0		As above
5. Was the sample size sufficiently large to provide confidence in the findings?	1	0		period, no power calculation but numbers comparable with similar studies.	1	0		As above
6. Was the test/service/intervention clearly described and delivered consistently across the study population?	1	0		Adequate description of service model available	1	0		The service model described and expected to be broadly the same across the sites
7. Were the outcome measures prespecified, clearly defined, valid, reliable, and assessed consistently across all study participants?	0	0	CD	Grossly inadequate description of data and methods	0	0	CD	Unclear from data presented
8. Were the people assessing the outcomes blinded to the participants' exposures/interventions?	0	0	CD	Grossly inadequate description of data and methods	0	1		Not possible to blind
9. Was the loss to follow-up after baseline 20% or less? Were those lost to follow-up accounted for in the analysis?	0	0	CD	Inadequate description of reasons for missing data	0	0	CD	Unclear from data presented
10. Did the statistical methods examine changes in outcome measures from before to after the intervention? Were statistical tests done that provided p values for the pre-to-post changes?	0	0	CD	Some statistical tests conducted but with grossly inadequate description of data and methods	0	0	CD	Unclear from data presented
11. Were outcome measures of interest taken multiple times before the intervention and multiple times after the intervention (i.e., did they use an interrupted time-series design)?	0	1			0	1		Outcome measures were not taken before or after in this way
12. If the intervention was conducted at a group level (e.g., a whole hospital, a community, etc.) did the statistical analysis take into account the use of individual-level data to determine effects at the group level?	0	1		Nil sub-analysis	0	1		No evidence of this

**Quality rating**	**Initials**	**Rating**
Rater #1	SP	Poor
Rater #2	GH	Poor

Additional Comments (If POOR, please state why): The report fails to provide adequate description of the methods used, and description data is incomplete.

* CD, cannot determine; NA, not applicable; NR, not reported

CASP Qualitative Checklist

Study: **Barnett K, Guiver N, Cheok F. Evaluation of the Three Community Rehabilitation Centres: FINAL REPORT. In: Health S, editor. South Australia: SA Health; 2011.**

	RATER 1				RATER 2			
**Criteria**	**Yes**	**Can't tell**	**No**	**Justification**	**Yes**	**Can't tell**	**No**	**Justification**
**1. Was there a clear statement of the aims of the research?** Consider: (1) what were the goals of the research; (2) why was it thought important; (3) its relevance.	0	0	1	Not of relevance to the qualitative data presented	1	0	0	Clearly stated in term of reference
**2. Is a qualitative methodology appropriate?** Consider: (1) if the research seeks to interpret or illuminate the actions and / or subjective experiences of research participants; (2) is qualitative research the right methodology for addressing the research goal?	0	1	0	Methodology not adequately described	0	1	0	The methodology is not well described
**3. Was the research design appropriate to address the aims of the research?** Consider: if the researcher has justified the research design (e.g. have they discussed how they decided which method to use)?	0	1	0	Methodology not adequately described	0	1	0	As above
**4. Was the recruitment strategy appropriate to the aims of the research?** Consider: (1) if the researcher has explained how the participants were selected; (2) if they explained why the participants they selected were the most appropriate to provide access to the type of knowledge sought by the study; (3) if there are any discussions around recruitment (e.g. why some people chose not to take part).	0	1	0	Methodology not adequately described	0	1	0	As above
**5. Was the data collected in a way that addressed the research issue?** Consider: (1) if the setting for data collection was justified; (2) if it is clear how data were collected (e.g. focus group, semi-structured interview etc); (3) if the researcher has justified the methods chosen; (4) if the researcher has made the methods explicit (e.g. for interview method, is there an indication of how the interviews were conducted, or did they use a topic guide); (5) if the methods were modified during the study. If so has the researcher explained how and why; (6) if the form of data is clear (e.g. tape recordings, video materials, notes); (7) if the researcher has discussed saturation of the data.	0	1	0	Methodology not adequately described	0	1	0	As above
**6. Has the relationship between the researcher and the participants been adequately considered?** Consider: (1) if the researcher critically examined their own role, potential bias and influence during formulation of the research questions an data collection (including sample selection and choice of location); (2) how the researhcer responds to events during the study and whether they considered the implications of any changes in the research design.	0	1	0	Methodology not adequately described	0	1	0	As above
**7. Have ethical issues been taken into consideration?** Consider: (1) is there sufficient details of how the research was explained to participants for the reader to assess whether ethical standards were maintained; (2) if the reseacher has discussed issues raised by the study (e.g. issues around informed consent or confidentiality or how they have handled the effects of the study on the participants during and after the study); (3) if approval has been sought from the ethics committee.	0	1	0	Methodology not adequately described	0	1	0	As above
**8. Was the data analysis sufficiently rigorous?** Consider: (1) if there is an in-depth description of the analysis process; (2) if thematic analysis is used. If so, is it clear how the categories/themes were derived from the data?; (3) whether the researcher explains how the data presented were selected from the original sample to demonstrate the analysis process; (4) if sufficent data are presented to support the findigns; (5) to what extent contradictory data are taken into account; (6) whether the researcher critically examined their own role, potential bias and influence during the analysis and selection of data for presentation.	0	1	0	Methodology not adequately described	0	1	0	As above
**9. Is there a clear statement of the findings?** Consider: (1) if the findings are explicit; (2) if there is adequate discussion of the evidence both for and against the researchers argument; (3) if the researcher has discussed the credibility of their findings (e.g. triangulation, respondent validation, more than one analyst); (4) if the findings are discussed in relation to the original research question.	0	1	0	Inadequate description	0	1	0	As above
**10. How valuable is the research?** Consider: (1) if the researcher discusses the contribution the study makes to existing knowledge or understanding e.g. do they consider the findings in relation to current practice or policy, or relevant research-based literature?; (2) if they identify new areas where research is necessary; (3) if the researchers have discussed whether or how the findings can be transferred to other populations or considered ways the research may be used.	0	1	0	Value limited by lack of detail regarding methodology.	0	1	0	As above

**ADDITIONAL RATING: Quality rating**	**Initials**	**Rating**
Rater #1	SP	Poor
Rater #2	GH	Poor

Additional Comments (If LOW, please state why): The absence of description of the methodology with regards to the qualitative data reported undermines the trustworthiness of conclusions drawn.

NIHL Checklist - Quality Assessment Tool for Case Series Studies

Study: **Chopra P, Herrman HE. The long-term outcomes and unmet needs of a cohort of former long-stay patients in Melbourne, Australia. Community mental health journal. 2011;47(5):531-41.**

	RATER 1				RATER 2			
Criteria	Yes	No	Other (CD, NR, NA)*	Justification	Yes	No	Other (CD, NR, NA)*	Justification
1. Was the study question or objective clearly stated?	1	0		Abstract states: "This study assessed the long-term outcomes for the original cohort of 18 residents of the Footbridge Community Care Unit (CCU), a residential psychiatric rehabilitation unit at St Vincent's Mental Health Melbourne." Objectives are defined clearly, but the ability of the design to mee the stated primary objectivng of assessing the long-term outcome of the initial cohort is less clear. The design permits consideration of post-CCU functioning but not the outcome achieved through engagement in CCU care.	1	0		Three fold objectives clearly stated'To assess long term outcomes…using clinician and self-report measures..identify unmet needs..develop recommendations for long-stay patients'
2. Was the study population clearly and fully described, including a case definition?	0	1		Some relevant details including gender are omitted. Measures of central tendency are inconsistently reported (mean vs median +/- range, and there is no documentation of SE/SD). No details are provided about the presence / absence of community treatment order. Additional detail around medication including CPZ dose equivalence would be informative.	0	0	CD	Sample was the 18 initial patients who were settled in CCU upon its opening in 1995. 14/18 patients (2 deaths, 2 did not consent) to the prospective part of the interview. There are some differences in the population i.e a range of duration of contact with service and type of co-morbidities
3. Were the cases consecutive?	1	0		Indicates full capture of the initial cohort (therefore by implication consequetive entry) for the quantitative component.	0	0		They are from the original cohort of residents at this CCU
4. Were the subjects comparable?	0	0	CD	See Criterion 2 re omitted details. Also characteristics of those consumers having adverse outcomes (e.g. incarceration, death or forensic care) were not compared to those who did not. Small sample size may have made such detail inappropriate (due to identifiability).	0	0	CD	The population is small and with some large difference i.e a range of duration of contact with service and type of co-morbidities i.e. ID, medical problems, substance misuse. There is no mention of the type of medications or dosages they were prescribed
5. Was the intervention clearly described?	1	0		Adequate description of CCU intervention is provided and detailed efforts to explore interventional aspects through the chart review process is evident.	1	0		There is considerable detail about the origins of Footbridge CCU, staffing, interventions provided, including how the model of service has changed over the years into more short and medium term rehabilitation rather than long term.
6. Were the outcome measures clearly defined, valid, reliable, and implemented consistently across all study participants?	1	0		Completed by the primary author at the time of the follow-up interview (consistent but unlinded rater), for 14/18. Those who could not be interviewed the most recent assessment was utilised. Both routine outcome measures chosen have good reliability and validity.	1	0		Medical record review assessed according to a set, standardised template. LSP/HONOS are widely used and validated tools. COLI used tape recorded interviews and transcription for accuracy.
7. Was the length of follow-up adequate?	1	0		Adequate duration and completeness.	1	0		Followup after 8 years allowed time for meaningful assessment of the patients after their discharge from CCU
8. Were the statistical methods well-described?	0	0		Descriptive statistics only.	0	1		Statistical analysis not mentioned or reported in the results, no p values. There was no description of how the themes from the COLI interviews/Qualitative aspect of study were decided on in a systematic way by the researchers.
9. Were the results well-described?	1	0		Yes but only at the descriptive level.	1	0		Yes there is adequate description of the results, but without the statistical analyses

Quality rating	Initials	Rating
Rater #1	SP	Fair
Rater #2	GH	Fair

Additional Comments (If LOW/POOR, please state why):

* CD, cannot determine; NA, not applicable; NR, not reported

CASP Qualitative Checklist

Study: **Chopra P, Herrman HE. The long-term outcomes and unmet needs of a cohort of former long-stay patients in Melbourne, Australia. Community mental health journal. 2011;47(5):531-41.**

	RATER 1				RATER 2			
**Criteria**	**Yes**	**Can't tell**	**No**	**Justification**	**Yes**	**Can't tell**	**No**	**Justification**
**1. Was there a clear statement of the aims of the research?** Consider: (1) what were the goals of the research; (2) why was it thought important; (3) its relevance.	1	0	0	Assessment of unmet needs defined as the objective (implicitly related to the qualitative component). Adequate contextual information provided to justify the relevance of this goal.	1	0	0	Objectives clearly stated and contextualised with reference to existing literature
**2. Is a qualitative methodology appropriate?** Consider: (1) if the research seeks to interpret or illuminate the actions and / or subjective experiences of research participants; (2) is qualitative research the right methodology for addressing the research goal?	1	0	0	Use of a pre-existing and validated semi-structured interview is appropriate for the stated purpose. The domains covered by this interview have direct relevance to the question posed.	1	0	0	Using a semi-structured interview tool (COLI) with domains to gather relevant qualitative data around psychosocial functioning, life satisfaction etc was appropriate to the aims of the research, and the COLI is described in detail in the article
**3. Was the research design appropriate to address the aims of the research?** Consider: if the researcher has justified the research design (e.g. have they discussed how they decided which method to use)?	0	1	0	Justification for the research design provided with reference to the appropriateness of a semi-structured interview given problems with initiating narrative description in the participant group anticipated. Theorerical orienation not addressed. Cohart review process and quantitative measures create opportunity for triangulation.	1	0	0	As above.
**4. Was the recruitment strategy appropriate to the aims of the research?** Consider: (1) if the researcher has explained how the participants were selected; (2) if they explained why the participants they selected were the most appropriate to provide access to the type of knowledge sought by the study; (3) if there are any discussions around recruitment (e.g. why some people chose not to take part).	1	0	0	Limited sample pool with efforts towards exhaustive sampling described. No description of the characteristics or reason for refusal for the 2/16 who declined. This is a limitation but given 87.5% of relevant and extant potential participants are included this is not a major concern.	1	0	0	Pragmatic/Convenience sample of almost all eligible participants other than those who passed away. No elaboration on why content unable to be obtained from 2 participants
**5. Was the data collected in a way that addressed the research issue?** Consider: (1) if the setting for data collection was justified; (2) if it is clear how data were collected (e.g. focus group, semi-structured interview etc); (3) if the researcher has justified the methods chosen; (4) if the researcher has made the methods explicit (e.g. for interview method, is there an indication of how the interviews were conducted, or did they use a topic guide); (5) if the methods were modified during the study. If so has the researcher explained how and why; (6) if the form of data is clear (e.g. tape recordings, video materials, notes); (7) if the researcher has discussed saturation of the data.	0	1	0	No detail provided about the setting in which the semi-structured interviews took place. Clear description of data collection methods with justification. Described use of the COLI makes the methods explicit. No report re modification whilst in progress. Form of data well described. Issues of saturation not discused.	0	1	0	Limited information about how COLI was carried out and some lack of description around methodology of how conclusions were drawn
**6. Has the relationship between the researcher and the participants been adequately considered?** Consider: (1) if the researcher critically examined their own role, potential bias and influence during formulation of the research questions an data collection (including sample selection and choice of location); (2) how the researhcer responds to events during the study and whether they considered the implications of any changes in the research design.	0	0	1	Not addressed. Role of the interviewer (Psychiatrist) in prior care of patients not considered, as well as the potential bias introduced by a clinical interviewer working within the system under investigation.	0	0	1	Not discussed, there is potential for a power imbalance and bias if a member of the service carried out the interviews, potentially suppressing more negative viewpoints
**7. Have ethical issues been taken into consideration?** Consider: (1) is there sufficient details of how the research was explained to participants for the reader to assess whether ethical standards were maintained; (2) if the reseacher has discussed issues raised by the study (e.g. issues around informed consent or confidentiality or how they have handled the effects of the study on the participants during and after the study); (3) if approval has been sought from the ethics committee.	0	1	0	Ethical clearance documented. No documentation around discussion of issues raised with participants. No description of detail provided to participants about the nature of participation.	0	1	0	Ethical clearance and informed consent provided by participants, however no further detail other than this provided about how impact of research explained to patients, issues around confidentiality etc.
**8. Was the data analysis sufficiently rigorous?** Consider: (1) if there is an in-depth description of the analysis process; (2) if thematic analysis is used. If so, is it clear how the categories/themes were derived from the data?; (3) whether the researcher explains how the data presented were selected from the original sample to demonstrate the analysis process; (4) if sufficent data are presented to support the findngs; (5) to what extent contradictory data are taken into account; (6) whether the researcher critically examined their own role, potential bias and influence during the analysis and selection of data for presentation.	0	1	0	Relatively brief description (context of mixed methods paper). No reporting of who or how many researchers were involved in the coding. No description of how / why extracts were chosen but with adequate illustration of themes present. Nil critical examination of the researchers own role and potential bias (and efforts to protect from this). Limitations in qualitative analysis not adequately considered.	0	1	0	There is very brief discussion around the the data analysis, and the role of the researchers and potential biases.
**9. Is there a clear statement of the findings?** Consider: (1) if the findings are explicit; (2) if there is adequate discussion of the evidence both for and against the researchers argument; (3) if the researcher has discussed the credibility of their findings (e.g. triangulation, respondent validation, more than one analyst); (4) if the findings are discussed in relation to the original research question.	1	0	0	Findings are made explicit. Limited consideration of evidence against findings. Triangulation supported by mixed methods approach. Related back to the original research question.	1	0	0	The findings are clearly stated and elaborated in the discussion section. There is little discussion about evidence against the researcher's argument. The findings are discussed in relation to the original research question
**10. How valuable is the research?** Consider: (1) if the researcher discusses the contribution the study makes to existing knowledge or understanding e.g. do they consider the findings in relation to current practice or policy, or relevant research-based literature?; (2) if they identify new areas where research is necessary; (3) if the researchers have discussed whether or how the findings can be transferred to other populations or considered ways the research may be used.	1	0	0	Well contextualised.	1	0	0	There is detailed discussion around this in the final section of the paper.

**ADDITIONAL RATING: Quality rating**	**Initials**	**Rating**
Rater #1	SP	Poor
Rater #2	GH	Poor

Additional Comments (If LOW/POOR, please state why): Key issues arising are the lack of consideration of reflexivity and description of the analytic process.

NIHL Checklist - Quality Assessment Tool for Observational Cohort and Cross-Sectional Studies

Study: **Farhall J, Trauer T, Newton R, Cheung P. Minimizing adverse effects on patients of involuntary relocation from long-stay wards to community residences. Psychiatr Serv. 2003;54(7):1022-7.**

	RATER 1				RATER 2			
**Criteria**	**Yes**	**No**	**Other (CD, NR, NA)***	**Justification**	**Yes**	**No**	**Other (CD, NR, NA)***	**Justification**
1. Was the research question or objective in this paper clearly stated?	1	0		Abstract states intention to examine the incidence of, and variables associated with, relocation trauma among 85 patients who moved from long-stay psychiatric wards to CCUs as part of a hospital closure. Introduction outlines five expectations stated but without designation of primary focus: (1) favouring community living; (2) CCU as a less restrictive environment; (3) relocation trauma only affecting a minority; (4) preparation at a transitional unit would reduce relocation trauma; (5) more transition preparation would reduce the likelihood of relocation trauma.	1	0		[This study] aimed to determine whether patients experienced relocation trauma and whether adjustment in the first month after the move was associated with the transition process' (Relocation trauma was defined as specific changes on PANSS and SOAS scores)
2. Was the study population clearly specified and defined?	1	0		87 patients transitioning from long-stay care. Demographics clearly detailed in table 1 with relevant considerations documented. By definition population is free of the key outcome considered.	1	0		It a convenience sample of 87 people transitioning from hospital to CCUs. Demographic tables provided. They were complex and have high rates of co-morbidity
3. Was the participation rate of eligible persons at least 50%?	1	0		Exhaustive sampling, all relevant cases included.	1	0		All 87 patients transitioning were included
4. Were all the subjects selected or recruited from the same or similar populations (including the same time period)? Were inclusion and exclusion criteria for being in the study prespecified and applied uniformly to all participants?	0	0	CD	Duration of mental illness differed but all sourced in the same context. No reporting of assessment of potential confounds impacting on relocation trauma as predictors (e.g. age, LOS). Also there is significant missing data across the measures considered and relocation trauma was only calculable for 78 of the original 85 who were transferred. Discussion around missing data not provided.	0	0	CD	There is some variation in the sample. Unclear what the admission criteria for the CCU was. Some missing data
5. Was a sample size justification, power description, or variance and effect estimates provided?	0	0	NR	Not reported. Potentially problematic given small effects described in previous literature.	0	0	NR	Don't see this anywhere
6. For the analyses in this paper, were the exposure(s) of interest measured prior to the outcome(s) being measured?	1	0		Exposure was the relocation event, this occurred prior to outcome assessment for included participants.	1	0		Yes outcomes measured one month after move. Differences between amount of preparation and site visits some participants obtained however prior to their final move, so the intensity of 'exposure' was not consistent and might have been mitigated by pre-preparation
7. Was the timeframe sufficient so that one could reasonably expect to see an association between exposure and outcome if it existed?	1	0		Expected timeframe for relocation trauma emergence not speficied in the literature review (though the timeframe for resolution was). However, the 1/12 pre-post timeframe is appropriate and consistent with the relevant literature.	1	0		One month between pre-and post move, 'relocation trauma' is more likely to occur in early stages of relocation however this is an assumption. I not however that some had more preparation and site visits than others which may have reduced their trauma
8. For exposures that can vary in amount or level, did the study examine different levels of the exposure as related to the outcome (e.g., categories of exposure, or exposure measured as continuous variable)?	0	0	NA	Defined exposure, consistently occuring. Aspects of the relocation environment were also assessed.	1	0		Yes they classified differently - 'shorter preparation', 'longer' preparation, 'five visits or less' and 'six visits or more' - but as a bimodal not continuous variable
9. Were the exposure measures (independent variables) clearly defined, valid, reliable, and implemented consistently across all study participants?	1	0		See 8.	0	1		There were differences between CCUs ie some transitional v 'permanent' - so these likely had different models of service
10. Was the exposure(s) assessed more than once over time?	0	0	NA	See 8.	0	1		Only one one month followup interview
11. Were the outcome measures (dependent variables) clearly defined, valid, reliable, and implemented consistently across all study participants?	1	0		Clearly defined and relevant. Prioritisation not documented.	0	0	CD	Incomplete data (81 out of 85 only having complete data), Relocation trauma status and transition preparation data for 77/87). PANSS and SOAS are valid instruments
12. Were the outcome assessors blinded to the exposure status of participants?	0	1		No control condition, no blinding possible given the nature of the investigation.	0	0	NR	No mention of blinding, not randomly allocation to CCU or to receive preparatory visits
13. Was loss to follow-up after baseline 20% or less?	1	0			1	0		
14. Were key potential confounding variables measured and adjusted statistically for their impact on the relationship between exposure(s) and outcome(s)?	1	0		Multple illness severity related variables considered.	1	0		Relocation trauma and severity of disorder were considered and calculated (but was not statistically significant)

**Quality Rating (Good, Fair, or Poor) (see guidance)**	**Initials**	**Rating**
Rater #1	SP	Good
Rater #2	GH	Good

Additional Comments (If POOR, please state why):

*CD, cannot determine; NA, not applicable; NR, not reported

NIHL Checklist - Quality Assessment Tool for Before-After (Pre-Post) Studies with no control group

Study: **Hamden A, Newton R, McCauley-Elsom K, Cross W. Is deinstitutionalization working in our community? International journal of mental health nursing. 2011;20(4):274-83.**

	RATER 1				RATER 2			
**Criteria**	**Yes**	**No**	**Other (CD, NR, NA)***	**Justification**	**Yes**	**No**	**Other (CD, NR, NA)***	**Justification**
1. Was the study question or objective clearly stated?	0	1		Stated aim: "Trauer et al. (2001) also discussed the need for further studies to look at the function of CCU and their emerging needs now that there is a significant reduction in the availability of inpatient beds, and this is the motivation behind this research." But the abstract describes the foucs as examining the impact of de-institutionalisation on consumers admitted to a regional community care unit between 1996-2007, and look at LOS and re-admission to acute care and the impact this might have on quality of life. Specific hypotheses are not clearly stated.	0	0	CD	Quality of life outcomes and and other aims ('1 attitude of current and past residents. Of CCU 2 if there is a reduction in LOS in CCU 4 the impact on readmissions to hospital) These 4 aims are stated in Methods section however no clear hypothesis. They referenced in literature search that other studies found high QOL scores but no change in symptoms after periods in rehabilitation, but unclear what they were expecting
2. Were eligibility/selection criteria for the study population prespecified and clearly described?	0	0	NR	Convenience sample, with two identified cohorts, current CCU residents (n=16) and a group of former residents identified through the client management interface. No specific statement re inclusion or exclusion criteria.	0	0	CD	Convenience sample of current CCU residents (n=16) and residents who were discharged between between 1996 - 2007 (n=15). No specific inclusion or exclusion criteria specified. Some demographic criteria on these residents (age, sex, location, diagnosis, LOS in CCU, readmissions to acute care) provided later in article)
3. Were the participants in the study representative of those who would be eligible for the test/service/intervention in the general or clinical population of interest?	0	0	CD	Good proportionate representation of the current consumer group (16/20). Insufficient description of the former and current CCU cohorts, including omission of diagnostic information and age and gender comparisons. No description provided for the rationale for presenting combined data. No detail provided about the characteristics of consumers who did not consent or were unable to be followed up to establish representativeness. Small response rate of former residents.	0	0	CD	They specified consumers with Severe Mental Illness, and said a retrospective examination of medical records was done to determine demographic data, however no detail provided ai.e. Gender, diagnosis, medical in CPZ equivalents. Table 1 shows double the number of males than females (81 v 40)
4. Were all eligible participants that met the prespecified entry criteria enrolled?	1	0		Regarding the LOS and re-admission data this was available from the administrative database for all consumers (including those not providing consent or not able to be followed up for qualitative interview.	0	0	CD	The criteria was either to be a current or ex-CCU resident. There were 101 potential ex residents but only 15 ended up being recruited due to complexities in the CMI system, where system not updated and residents had out of date contact details. 16 out of 20 current CCU residents provided informed consent, did not explain why 4 did not provide consent
5. Was the sample size sufficiently large to provide confidence in the findings?	0	0	CD	No reporting of power calculation to detect anticipated differences between or within groups. Small group size for	0	0	CD	Small sample size with low respnse rate so hard to draw firm conclusions, no power
6. Was the test/service/intervention clearly described and delivered consistently across the study population?	0	0	CD	Insufficient detail provided to establish the equivalence or points of difference between the nature of CCU care delivered to current and former CCU consumers.	0	0	CD	All were either residents or ex-residents of the same CCU, but it may have been operational changes, changes to model of service, or improvements in the interventions provided between 1996 to 2007 and this was not adequately described
7. Were the outcome measures prespecified, clearly defined, valid, reliable, and assessed consistently across all study participants?	0	0	CD	Justification is provided for both PAQ and MANSA - both have face validity in assessing the target outcomes / constructs. However the planned analysis based on these measures was not decribed (e.g. the primary focus and secondary considerations given multiple components to each measure). Information about LOS calculation method and periods are inadequately defined.	0	0	CD	PAQ, MANSA are declared as valid and reliable instruments and statistics provided. CMI database is unreliable to assess LOS/readmissions as data missing if patient had moved and had contact with another service for example.
8. Were the people assessing the outcomes blinded to the participants' exposures/interventions?	0	0	NR	No information about blinding reported.	0	1		Can't blind, everyone was in the same CCU
9. Was the loss to follow-up after baseline 20% or less? Were those lost to follow-up accounted for in the analysis?	1	0		Retrospective administrative data for the quantitative measure, therefore no loss to follow-up detailed.	1	0		They looked through medical records retrospectively
10. Did the statistical methods examine changes in outcome measures from before to after the intervention? Were statistical tests done that provided p values for the pre-to-post changes?	0	0	CD	Inconsistent application of statistical tests of significance without clear justification, additionally inadequate information provided about the decision to combine or separate groups on individual outcomes.	0	0	CD	They compared discharged v current residents and their outcome measures. Qualitative data in MANSA and PAQ was not subject to statistical test. Tests of significance for quantitative data i.e. LOS/readmission rates did not indicate statistical significance (p values)
11. Were outcome measures of interest taken multiple times before the intervention and multiple times after the intervention (i.e., did they use an interrupted time-series design)?	0	0	CD	Inadequate detail provided in relation to the process of calculation of LOS and readmissiond data.	0	0	CD	No the outcome measures were not calculated before admission to CCU for current residents, and for CCU residents that were discharged only aft er they left CCU
12. If the intervention was conducted at a group level (e.g., a whole hospital, a community, etc.) did the statistical analysis take into account the use of individual-level data to determine effects at the group level?	0	1		No evidence of adjustment (also differential outcome between current and former residents not considered).	0	0	NR	I don't see this reported here. The two groups were likely to have significant difference i.e. some were still CCU residents, some discharged after some time at CCU, so would have been at different points in their rehabilitation, however this does not appear to be accounted for in the results

**Quality rating**	**Initials**	**Rating**
Rater #1	SP	Poor
Rater #2	GH	Poor

Additional Comments (If POOR, please state why): Key issues included the adequacy of methodological reporting, sample selection and analytic approach.

* CD, cannot determine; NA, not applicable; NR, not reported

NIHL Checklist - Quality Assessment Tool for Observational Cohort and Cross-Sectional Studies

Study: **Hobbs, C, Newton, L., Tennant, C., Rosen, A. & Tribe, K. (2002) Deinstitutionalization for Long-Term Mental Illness: A 6-Year Evaluation. ANZJP, 35: 60.**

	RATER 1				RATER 2			
**Criteria**	**Yes**	**No**	**Other (CD, NR, NA)***	**Justification**	**Yes**	**No**	**Other (CD, NR, NA)***	**Justification**
1. Was the research question or objective in this paper clearly stated?	1	0		Multiple objectives are detailed and the paper represents a summation of previous work. A greater degree of specification could be provided, but adequate detail is given in the associated publications. "The objective of the 6-year follow-up was threefold: (a) to determine the accommodation style and level of care required by residents; (b) to evaluate clinical changes over time; and (c) to gain the residents' perspectives of their lives. To this end the 6-year evaluation included both quantitative and qualitative components." (p61)	0	0	CD	This is a 6 year evaluation post discharge of patients transitioned to community residential care with a rehab focus. The objectives were to '1. Determine the accomodation style and level of care required 2. Evaluate clinical changes over time 3. to gain perspective of their lives' These are not clearly focussed questions, more explorative in nature
2. Was the study population clearly specified and defined?	1	0		Adequate description is provided in the references sub-ordinate paper.	1	0		Detail on demographics referenced an earlier study, with diagnosis, duration of illness, age at discharge, all were considered not suitable for discharge if the hospital were not forced to close
3. Was the participation rate of eligible persons at least 50%?	1	0		Convenience sample, exhuastive of the specific context under investigation.	1	0		All 40 patients that were transitioning were included
4. Were all the subjects selected or recruited from the same or similar populations (including the same time period)? Were inclusion and exclusion criteria for being in the study prespecified and applied uniformly to all participants?	1	0		De-institutionalisation cohort.	1	0		Sample was a group of 40 patients who were recruited from a single hospital. (?Convenience sample)
5. Was a sample size justification, power description, or variance and effect estimates provided?			NA	Convenience sample, exhuastive of the specific context under investigation.	0	0	NR	
6. For the analyses in this paper, were the exposure(s) of interest measured prior to the outcome(s) being measured?			NA	Obervational cohort, shared exposure (transition to community residence).	1	0		The exposure was the relocation event, The four community residential facilities were described as similar
7. Was the timeframe sufficient so that one could reasonably expect to see an association between exposure and outcome if it existed?	1	0		6 years represents an adequate time period to explore post-transition adjustment.	1	0		6 year evaluation post relocation is appropriate to assess how patients respond to community rehabilitation environment
8. For exposures that can vary in amount or level, did the study examine different levels of the exposure as related to the outcome (e.g., categories of exposure, or exposure measured as continuous variable)?			NA	See 6.	0	0	NR	
9. Were the exposure measures (independent variables) clearly defined, valid, reliable, and implemented consistently across all study participants?			NA	See 6.	0	0	NA	Relocation to community residential care with rehab interventions is a defined exposure consistently occurring
10. Was the exposure(s) assessed more than once over time?			NA	See 6.	0	0	NA	
11. Were the outcome measures (dependent variables) clearly defined, valid, reliable, and implemented consistently across all study participants?			CD	These are defined and justified. Non blinded assesments, no specific consideration of inter-rater reliability within study.	1	0		Clearly defined and relevant. BPRS, LSP, MADRS, QOL are validated tools.
12. Were the outcome assessors blinded to the exposure status of participants?		1		See 11.	0	0	NA	Not applicable to this study
13. Was loss to follow-up after baseline 20% or less?			NA		0	1		No, 6 were in hospital at 6 year followup and 3 had died
14. Were key potential confounding variables measured and adjusted statistically for their impact on the relationship between exposure(s) and outcome(s)?			CD	Utility of the comparison of community and hospital based residents at follow-up is limited by the small number of hospital based residents.	0	0	CD	No confounding variables identified or statistical adjustment for same. There is commentary about incidents, readmissions that occurred over the 6 years (no alcohol or substance abuse issues arose!)

**Quality Rating (Good, Fair, or Poor) (see guidance)**	**Initials**	**Rating**
Rater #1	SP	Fair
Rater #2	GH	Fair

Additional Comments (If POOR, please state why):

*CD, cannot determine; NA, not applicable; NR, not reported

CASP Qualitative Checklist

Study: **Hobbs, C, Newton, L., Tennant, C., Rosen, A. & Tribe, K. (2002) Deinstitutionalization for Long-Term Mental Illness: A 6-Year Evaluation. ANZJP, 35: 60.**

	RATER 1				RATER 2			
Criteria	Yes	Can't tell	No	Justification	Yes	Can't tell	No	Justification
**1. Was there a clear statement of the aims of the research?** Consider: (1) what were the goals of the research; (2) why was it thought important; (3) its relevance.	0	0	1	Inadequately defined		1		Domains of qualitative semi structured interviews are briefly mentioned but no detail provided about reasons for inclusion, though appear relevant for the purpose of the research question
**2. Is a qualitative methodology appropriate?** Consider: (1) if the research seeks to interpret or illuminate the actions and / or subjective experiences of research participants; (2) is qualitative research the right methodology for addressing the research goal?	0	1	0	Inadequately defined. Note that one of the earlier studies reported on in this paper describes an extensive ethnographic approach to data collection. However, even in this paper minimal information is provided in relation to the analytic approach.		1		No information provided and no analysis in article. Semi structured interviews would be appropriate to investigate domains that are relevant to the aims of the research
**3. Was the research design appropriate to address the aims of the research?** Consider: if the researcher has justified the research design (e.g. have they discussed how they decided which method to use)?	0	1	0	Inadequately defined		1		Not clear
**4. Was the recruitment strategy appropriate to the aims of the research?** Consider: (1) if the researcher has explained how the participants were selected; (2) if they explained why the participants they selected were the most appropriate to provide access to the type of knowledge sought by the study; (3) if there are any discussions around recruitment (e.g. why some people chose not to take part).	0	1	0	Inadequately defined		1		As above
**5. Was the data collected in a way that addressed the research issue?** Consider: (1) if the setting for data collection was justified; (2) if it is clear how data were collected (e.g. focus group, semi-structured interview etc); (3) if the researcher has justified the methods chosen; (4) if the researcher has made the methods explicit (e.g. for interview method, is there an indication of how the interviews were conducted, or did they use a topic guide); (5) if the methods were modified during the study. If so has the researcher explained how and why; (6) if the form of data is clear (e.g. tape recordings, video materials, notes); (7) if the researcher has discussed saturation of the data.	0	1	0	Inadequately defined		1		Results of qualitative interviews not described in article
**6. Has the relationship between the researcher and the participants been adequately considered?** Consider: (1) if the researcher critically examined their own role, potential bias and influence during formulation of the research questions an data collection (including sample selection and choice of location); (2) how the researhcer responds to events during the study and whether they considered the implications of any changes in the research design.	0	0	1	Not defined		1		No clear from the article
**7. Have ethical issues been taken into consideration?** Consider: (1) is there sufficient details of how the research was explained to participants for the reader to assess whether ethical standards were maintained; (2) if the reseacher has discussed issues raised by the study (e.g. issues around informed consent or confidentiality or how they have handled the effects of the study on the participants during and after the study); (3) if approval has been sought from the ethics committee.	0	1	0	Not defined		1		No clear from the article
**8. Was the data analysis sufficiently rigorous?** Consider: (1) if there is an in-depth description of the analysis process; (2) if thematic analysis is used. If so, is it clear how the categories/themes were derived from the data?; (3) whether the researcher explains how the data presented were selected from the original sample to demonstrate the analysis process; (4) if sufficent data are presented to support the findigns; (5) to what extent contradictory data are taken into account; (6) whether the researcher critically examined their own role, potential bias and influence during the analysis and selection of data for presentation.	0	0	1	Inadequately defined		1		Not clear from the article
**9. Is there a clear statement of the findings?** Consider: (1) if the findings are explicit; (2) if there is adequate discussion of the evidence both for and against the researchers argument; (3) if the researcher has discussed the credibility of their findings (e.g. triangulation, respondent validation, more than one analyst); (4) if the findings are discussed in relation to the original research question.	0	0	1	Inadequately defined		1		As above
**10. How valuable is the research?** Consider: (1) if the researcher discusses the contribution the study makes to existing knowledge or understanding e.g. do they consider the findings in relation to current practice or policy, or relevant research-based literature?; (2) if they identify new areas where research is necessary; (3) if the researchers have discussed whether or how the findings can be transferred to other populations or considered ways the research may be used.	0	0	1	Minimal presentation or discussion of qualitative data.			1	Unable to assess since no findings from qualitative aspect of the study presented in the article

**ADDITIONAL RATING: Quality rating**	Initials	Rating
Rater #1	SP	POOR
Rater #2	GH	POOR

Additional Comments (If LOW/POOR, please state why): Inadequate information is provided about the methodology to support the trustworthiness of reported findings

CASP Qualitative Checklist

Study: **McKenna, B., et al. (2016). "Recovery-Oriented Mental Health Practice in a Community Care Unit: An Exploratory Study." J Forensic Nurs 12(4): 167-175.**

	RATER 1				RATER 2			
**Criteria**	**Yes**	**Can't tell**	**No**	**Justification**	**Yes**	**Can't tell**	**No**	**Justification**
**1. Was there a clear statement of the aims of the research?** Consider: (1) what were the goals of the research; (2) why was it thought important; (3) its relevance.	1	0	0	"The aims of the study were to (a) describe what aspects of the current model of care fit within the nine domains of recovery in the current CCU and (b) describe the pragmatic processes that staff use to mold their care within the nine domains of recovery."	1	0	0	Clear and focussed question adequately contextualised - how current model of service fits within 9 domains of recovery in Victorian context
**2. Is a qualitative methodology appropriate?** Consider: (1) if the research seeks to interpret or illuminate the actions and / or subjective experiences of research participants; (2) is qualitative research the right methodology for addressing the research goal?	0	1	0	Design is described as 'exploratory,' however no guidance is provided as to the theoretical framework for the analysis beyond reference to 'thematic analysis' and a 'general inductive approach'. Some justification provided, but approach remains unclear.	0	1	0	Exploratory research which sought to overcome problem of lack of knowledge of recovery in CCUs, interviews with patients and staff was appropriate to investigate this problem and address the study question, rather than quantitative approach
**3. Was the research design appropriate to address the aims of the research?** Consider: if the researcher has justified the research design (e.g. have they discussed how they decided which method to use)?	0	1	0	See Item 3.	0	1	0	Some methodological flaws, in terms of how the interview schedule was developed and efforts to reduce bias
**4. Was the recruitment strategy appropriate to the aims of the research?** Consider: (1) if the researcher has explained how the participants were selected; (2) if they explained why the participants they selected were the most appropriate to provide access to the type of knowledge sought by the study; (3) if there are any discussions around recruitment (e.g. why some people chose not to take part).	0	1	0	No discussion of participants versus non-participants. No indication of overall potential samply pool from which the 21 consenters were derived.	0	1	0	21 Stakeholders, a minority of who (n=7) that were patients of the service, the remainder were 11 staff and 3 carers. People self selected into the study by responding to a flyer
**5. Was the data collected in a way that addressed the research issue?** Consider: (1) if the setting for data collection was justified; (2) if it is clear how data were collected (e.g. focus group, semi-structured interview etc); (3) if the researcher has justified the methods chosen; (4) if the researcher has made the methods explicit (e.g. for interview method, is there an indication of how the interviews were conducted, or did they use a topic guide); (5) if the methods were modified during the study. If so has the researcher explained how and why; (6) if the form of data is clear (e.g. tape recordings, video materials, notes); (7) if the researcher has discussed saturation of the data.	1	0	0	No discussion of data saturation. Inadequate description of the methods is provided regarding training, use of field notes etc.	1	0	0	There is adequate description and justification of how semi structured interviews were conducted and detail around data collection and analysis provided
**6. Has the relationship between the researcher and the participants been adequately considered?** Consider: (1) if the researcher critically examined their own role, potential bias and influence during formulation of the research questions an data collection (including sample selection and choice of location); (2) how the researhcer responds to events during the study and whether they considered the implications of any changes in the research design.	0	0	1	Reflexivity issues inadequately considered. No discussion of the exact nature of pre-existing relationships, nor of preconceptions brought to the project.	0	1	0	Mention of a consumer researcher being involved in conducting interviews, in an effort to reduce imbalanced power dynamic, however other relationship of researchers to staff and patients not clearly outlined
**7. Have ethical issues been taken into consideration?** Consider: (1) is there sufficient details of how the research was explained to participants for the reader to assess whether ethical standards were maintained; (2) if the reseacher has discussed issues raised by the study (e.g. issues around informed consent or confidentiality or how they have handled the effects of the study on the participants during and after the study); (3) if approval has been sought from the ethics committee.	0	0	1	Informed consent process followed. Ethics board approval not discussed. No detail of how sensitive information would be or was handled.	0	1	0	Informed consent obtained but no explicit mention of Ethical Approval prior to the commencement of the project
**8. Was the data analysis sufficiently rigorous?** Consider: (1) if there is an in-depth description of the analysis process; (2) if thematic analysis is used. If so, is it clear how the categories/themes were derived from the data?; (3) whether the researcher explains how the data presented were selected from the original sample to demonstrate the analysis process; (4) if sufficent data are presented to support the findings; (5) to what extent contradictory data are taken into account; (6) whether the researcher critically examined their own role, potential bias and influence during the analysis and selection of data for presentation.	0	1	0	Insufficient data presented to support the findings (though enough to build and maintain the narrative). No consideration of reflexivity issues beyond the acknowledgement of potential bias through dual roles. No description of how transcripts were chose.	0	1	0	There is description of themes and thematic analysis but lack of detail around how the themes were generated and how rigorous the methodology was
**9. Is there a clear statement of the findings?** Consider: (1) if the findings are explicit; (2) if there is adequate discussion of the evidence both for and against the researchers argument; (3) if the researcher has discussed the credibility of their findings (e.g. triangulation, respondent validation, more than one analyst); (4) if the findings are discussed in relation to the original research question.	0	1	0	Findings are clearly described. Evidence against the findings is not considered, as well as detailed acknowledgement of study limitations. Multiple raters used, no discussion of respondent validation. Findings are related back to the original question.	0	1	0	Findings are clearly stated
**10. How valuable is the research?** Consider: (1) if the researcher discusses the contribution the study makes to existing knowledge or understanding e.g. do they consider the findings in relation to current practice or policy, or relevant research-based literature?; (2) if they identify new areas where research is necessary; (3) if the researchers have discussed whether or how the findings can be transferred to other populations or considered ways the research may be used.	1	0	0	No discussion of transferability. Study does add new knowledge to an under researched area. Despite the limitations the findings add value given the limited work exploring recovery oriented practice. Key limitations relate to the lack of detailed description of the theoretical approach, methods and limited data presented in support of the analysis.No discussion of further research questions arising.	1	0	0	Contributes to an under researched area, and authors make particular references to challenges to generalising to other specialist settings (clinical forensic nursing), and explain limitations in using their conclusions to determine overall effectiveness of treatment in CCUs

**ADDITIONAL RATING: Quality rating**	**Initials**	**Rating**
Rater #1	SP	Fair
Rater #2	GH	Fair

Additional Comments (If LOW/POOR, please state why): NA

NIHL Checklist - Quality Assessment Tool for Observational Cohort and Cross-Sectional Studies

Study: **Meehan T, Stedman T, Parker S. Survey of consumers in Community and Hospital based residential rehabilitation programs in Queensland. nd**

	RATER 1				RATER 2			
**Criteria**	**Yes**	**No**	**Other (CD, NR, NA)***	**Justification**	**Yes**	**No**	**Other (CD, NR, NA)***	**Justification**
1. Was the research question or objective in this paper clearly stated?	0	0	CD	Broad intention clearly stated. However specific rationale for selection of measures for comparison not clear. How were the variables to be included / not-included slected.	0	0	CD	The objective is stated, however the basis for comparison i.e. the variables looked at are not justified
2. Was the study population clearly specified and defined?	1	0		Clear description of services, and these were placed in the context of the relevant literature.	1	0		The population are clearly defined
3. Was the participation rate of eligible persons at least 50%?	1	0		Administrative audit. 100% of data available for most variables with the exception of routine outcomes, CPZ equivalence and family contact data	1	0		Participation rate above 50% (it is an audit)
4. Were all the subjects selected or recruited from the same or similar populations (including the same time period)? Were inclusion and exclusion criteria for being in the study prespecified and applied uniformly to all participants?	1	0		See above.	1	0		
5. Was a sample size justification, power description, or variance and effect estimates provided?	0	0	CD	Exhaustive sampling but no consideration of power to detect differences based on the available sample.	0	0	CD	Don't see a power calculation
6. For the analyses in this paper, were the exposure(s) of interest measured prior to the outcome(s) being measured?	0	0	NA	No exposure, intention to establish the comparability of the groups.			NA	Not applicable
7. Was the timeframe sufficient so that one could reasonably expect to see an association between exposure and outcome if it existed?	0	0	NA				NA	As above
8. For exposures that can vary in amount or level, did the study examine different levels of the exposure as related to the outcome (e.g., categories of exposure, or exposure measured as continuous variable)?	0	0	NA				NA	As above
9. Were the exposure measures (independent variables) clearly defined, valid, reliable, and implemented consistently across all study participants?	0	0	NA				NA	As above
10. Was the exposure(s) assessed more than once over time?	0	0	NA				NA	As above
11. Were the outcome measures (dependent variables) clearly defined, valid, reliable, and implemented consistently across all study participants?	0	0	CD	No information reported on the training of raters beyond the site visits from benchmarking staff to ensure understandings of the information collection requirements. Reliance on administrative data systems, presumably multiple raters etc.			CD	
12. Were the outcome assessors blinded to the exposure status of participants?	0	1		No evidence of blinding, reliance on routine administrative data sets for outcome measures.		1		Not blinded
13. Was loss to follow-up after baseline 20% or less?	0	0	NA				NA	
14. Were key potential confounding variables measured and adjusted statistically for their impact on the relationship between exposure(s) and outcome(s)?	0	1	NR	Potential impact of patient-level characteristics on group level data not considered. Points of interest include comparison of consumers with shorter or longer length of stay (given the expected over-representation of long-stay), voluntary versus involuntary engagement etc on functional measures.		1		Insufficient exploration of confounding variables

**Quality Rating (Good, Fair, or Poor) (see guidance)**	**Initials**	**Rating**
Rater #1	DS	Fair
Rater #2	GH	Fair

Additional Comments (If POOR, please state why):

*CD, cannot determine; NA, not applicable; NR, not reported

CASP Qualitative Checklist

Study: **Meurk C, Parker S, Newman E, Dark F. Staff expectations of an integrated model of residential rehabilitation for people with severe and persisting mental illness: A pragmatic grounded theory analysis. The University of Queensland, Unpublished Manuscript, 2018.**

	**RATER 1**				**RATER 2**			
**Criteria**	**Yes**	**Can't tell**	**No**	**Justification**	**Yes**	**Can't tell**	**No**	**Justification**
**1. Was there a clear statement of the aims of the research?** Consider: (1) what were the goals of the research; (2) why was it thought important; (3) its relevance.	1	0	0	"This article analyses qualitative interviews undertaken with staff at two new CCUs trialling a staffing model incorporating peer support. The analysis considers staff understandings and expectations of recovery-oriented rehabilitation services in an Australian setting..."<br><br>The importance of this was contextualised with reference to trends towards increased emphasis on peer support roles, and the practical challenges associated with implementing these effectively.	1	0	0	The aim of the research is to examine how peer support workers have been implemented into the CCU model of service and both PSW and clinical staff's perspective on this, it is clearly outlined in introduction
**2. Is a qualitative methodology appropriate?** Consider: (1) if the research seeks to interpret or illuminate the actions and / or subjective experiences of research participants; (2) is qualitative research the right methodology for addressing the research goal?	1	0	0	Exploratory goal fits with the qualitative methods chosen.	1	0	0	"Qualitative semi-structured interviews were conducted with ten peer support workers and five clinical staff on commencement at Community Care Units that opened in 2014 and 2015", this approach is appropriate in order to gather information around the perspectives of staff, both peer support and clinical to address the aims of the research. No indication for quantitative approach.
**3. Was the research design appropriate to address the aims of the research?** Consider: if the researcher has justified the research design (e.g. have they discussed how they decided which method to use)?	0	1	0	Articulated in parent study protocol, and approach documented aligns with exploratory qualitative research methods of relevance to the stated aim. Do direct justification of the method chosen with reference to other approaches.	1	0	0	Semi-structured interviews with both clinical and PSW allow a multidimensional view of the issues and reduces bias, there is more detail in the protocol
**4. Was the recruitment strategy appropriate to the aims of the research?** Consider: (1) if the researcher has explained how the participants were selected; (2) if they explained why the participants they selected were the most appropriate to provide access to the type of knowledge sought by the study; (3) if there are any discussions around recruitment (e.g. why some people chose not to take part).	0	1	0	Did not use purposive sampling as would be typically employed in grounded theory. However, the approach was limited by the small potential sample pool. Iterative sampling to achieve saturation was used. Additionally, the consenting participants are nearly exhaustive of the potential pool.	0	1	0	Convenience sample of staff and consumers at two particular CCUs in Queensland, I note that purposive sampling is usually the methodology used in grounded theory
**5. Was the data collected in a way that addressed the research issue?** Consider: (1) if the setting for data collection was justified; (2) if it is clear how data were collected (e.g. focus group, semi-structured interview etc); (3) if the researcher has justified the methods chosen; (4) if the researcher has made the methods explicit (e.g. for interview method, is there an indication of how the interviews were conducted, or did they use a topic guide); (5) if the methods were modified during the study. If so has the researcher explained how and why; (6) if the form of data is clear (e.g. tape recordings, video materials, notes); (7) if the researcher has discussed saturation of the data.	1	0	0	Clear description provided in the study and parent study protocol of the data collection setting and approach. Interview schedule and method well articularted in published protocol.	1	0	0	This is well described in the study protocol and also in the article.
**6. Has the relationship between the researcher and the participants been adequately considered?** Consider: (1) if the researcher critically examined their own role, potential bias and influence during formulation of the research questions an data collection (including sample selection and choice of location); (2) how the researhcer responds to events during the study and whether they considered the implications of any changes in the research design.	0	1	0	This is explicitly explored in the parent study protocol but not in the paper itself.	0	1	0	I don't see the relationship between the researchers and staff explicitly mentioned in this particular article
**7. Have ethical issues been taken into consideration?** Consider: (1) is there sufficient details of how the research was explained to participants for the reader to assess whether ethical standards were maintained; (2) if the reseacher has discussed issues raised by the study (e.g. issues around informed consent or confidentiality or how they have handled the effects of the study on the participants during and after the study); (3) if approval has been sought from the ethics committee.	0	1	0	Ethical approval is documented. Ethical issues considered in the protocol. Issues arising during the study not documented.	0	1	0	The is ethical approval obtained, but because the researchers are clinically involved and managers within the CCU there is potential for biasing the results of the interviews. This isn't addressed in the article. Particularly when job security is a factor mentioned by the PSWs. This may bias the results of the interviews in a more positive way and suppress more negative views.
**8. Was the data analysis sufficiently rigorous?** Consider: (1) if there is an in-depth description of the analysis process; (2) if thematic analysis is used. If so, is it clear how the categories/themes were derived from the data?; (3) whether the researcher explains how the data presented were selected from the original sample to demonstrate the analysis process; (4) if sufficent data are presented to support the findigns; (5) to what extent contradictory data are taken into account; (6) whether the researcher critically examined their own role, potential bias and influence during analysis and selection of data for presentation.	1	0	0	Clear description is provided of the approach to the analysis. Multiple extracts provided in support of themes. Process of selection of illustrative extracts is documented. Reflexivity issues considered in published protocol.	1	0	0	The authors have rigorously analysed the data. They provide detail from the interviews to support the themes elicited.
**9. Is there a clear statement of the findings?** Consider: (1) if the findings are explicit; (2) if there is adequate discussion of the evidence both for and against the researchers argument; (3) if the researcher has discussed the credibility of their findings (e.g. triangulation, respondent validation, more than one analyst); (4) if the findings are discussed in relation to the original research question.	1	0	0	Findings clearly articulated and the relationship between themes is documented. Respondent verification was used.	1	0	0	Identification of 'success' and 'risk' factors, relationship to recovery principles, and implications for future service delivery outlined and supported by evidence form interviews.
**10. How valuable is the research?** Consider: (1) if the researcher discusses the contribution the study makes to existing knowledge or understanding e.g. do they consider the findings in relation to current practice or policy, or relevant research-based literature?; (2) if they identify new areas where research is necessary; (3) if the researchers have discussed whether or how the findings can be transferred to other populations or considered ways the research may be used.	0	1	0	Provides information relating to an unresearched topic. Case-to-case transferability not explicitly discussed but conclusion does draw out general prinicples of relevance to transfer.	1	0	0	The authors have contributed to an under researched area, particularly in a Queensland context, so it adds value to the literature. It is methodologically rigorous in many aspects.

**ADDITIONAL RATING: Quality rating**	**Initials**	**Rating**
Rater #1	DS	Fair
Rater #2	GH	Fair

Additional Comments (If LOW/POOR, please state why): NA

CASP Qualitative Checklist

Study: **Munro J, Palmada M, Russell A, Taylor P, Heir B, McKay J, et al. Queensland extended care services for people with severe mental illness and the role of occupational therapy. Australian occupational therapy journal. 2007;54:257-65**

	RATER 1				RATER 2			
**Criteria**	**Yes**	**Can't tell**	**No**	**Justification**	**Yes**	**Can't tell**	**No**	**Justification**
**1. Was there a clear statement of the aims of the research?** Consider: (1) what were the goals of the research; (2) why was it thought important; (3) its relevance.	0	1	0	Presentation of single consumer and carer cases as testimonials not well contextualised within the overall aim of the paper.	0	0	1	Aims are not well stated or defined
**2. Is a qualitative methodology appropriate?** Consider: (1) if the research seeks to interpret or illuminate the actions and / or subjective experiences of research participants; (2) is qualitative research the right methodology for addressing the research goal?	0	1	0	Nil description of qualitative methodology	0	0	1	Only verbatim statement of a carer, and consumer provided. No methodology explored
**3. Was the research design appropriate to address the aims of the research?** Consider: if the researcher has justified the research design (e.g. have they discussed how they decided which method to use)?	0	0	1	Nil description of qualitative methodology, single voice	0	0	1	No discussion of the methodology
**4. Was the recruitment strategy appropriate to the aims of the research?** Consider: (1) if the researcher has explained how the participants were selected; (2) if they explained why the participants they selected were the most appropriate to provide access to the type of knowledge sought by the study; (3) if there are any discussions around recruitment (e.g. why some people chose not to take part).	0	1	0	Nil description of qualitative methodology	0	0	1	As above
**5. Was the data collected in a way that addressed the research issue?** Consider: (1) if the setting for data collection was justified; (2) if it is clear how data were collected (e.g. focus group, semi-structured interview etc); (3) if the researcher has justified the methods chosen; (4) if the researcher has made the methods explicit (e.g. for interview method, is there an indication of how the interviews were conducted, or did they use a topic guide); (5) if the methods were modified during the study. If so has the researcher explained how and why; (6) if the form of data is clear (e.g. tape recordings, video materials, notes); (7) if the researcher has discussed saturation of the data.	0	1	0	Nil description of qualitative methodology	0	0	1	As above
**6. Has the relationship between the researcher and the participants been adequately considered?** Consider: (1) if the researcher critically examined their own role, potential bias and influence during formulation of the research questions an data collection (including sample selection and choice of location); (2) how the researhcer responds to events during the study and whether they considered the implications of any changes in the research design.	0	0	1	Nil description of qualitative methodology	0	0	1	As above
**7. Have ethical issues been taken into consideration?** Consider: (1) is there sufficient details of how the research was explained to participants for the reader to assess whether ethical standards were maintained; (2) if the reseacher has discussed issues raised by the study (e.g. issues around informed consent or confidentiality or how they have handled the effects of the study on the participants during and after the study); (3) if approval has been sought from the ethics committee.	0	0	1	Nil description of qualitative methodology	0	0	1	As above
**8. Was the data analysis sufficiently rigorous?** Consider: (1) if there is an in-depth description of the analysis process; (2) if thematic analysis is used. If so, is it clear how the categories/themes were derived from the data?; (3) whether the researcher explains how the data presented were selected from the original sample to demonstrate the analysis process; (4) if sufficent data are presented to support the findigns; (5) to what extent contradictory data are taken into account; (6) whether the researcher critically examined their own role, potential bias and influence during the analysis and selection of data for presentation.	0	0	1	Nil description to support analytic process	0	0	1	As above
**9. Is there a clear statement of the findings?** Consider: (1) if the findings are explicit; (2) if there is adequate discussion of the evidence both for and against the researchers argument; (3) if the researcher has discussed the credibility of their findings (e.g. triangulation, respondent validation, more than one analyst); (4) if the findings are discussed in relation to the original research question.	0	0	1	Inadequate	0	0	1	As above
**10. How valuable is the research?** Consider: (1) if the researcher discusses the contribution the study makes to existing knowledge or understanding e.g. do they consider the findings in relation to current practice or policy, or relevant research-based literature?; (2) if they identify new areas where research is necessary; (3) if the researchers have discussed whether or how the findings can be transferred to other populations or considered ways the research may be used.	0	0	1	Lack of methodological description limits value	0	0	1	As above

**ADDITIONAL RATING: Quality rating**	**Initials**	**Rating**
Rater #1	SP	Poor
Rater #2	GH	Poor

Additional Comments (If LOW/POOR, please state why):

Consumer and carer qualitative information is presented as as a testimonial, no description of the method through which these individuals came to be samples, the analytic process and of the relationships between the researchers and the respondents.

CASP Qualitative Checklist

Study: **Newton et al. (2000) Deinstitutionalisation for long-term mental illness: an ethnographic study. ANZJP**

	RATER 1				RATER 2			
Criteria	Yes	Can't tell	No	Justification	Yes	Can't tell	No	Justification
**1. Was there a clear statement of the aims of the research?** Consider: (1) what were the goals of the research; (2) why was it thought important; (3) its relevance.	0	0	1	Paper emphasises the relevance of qualitative research, and the goal of mirroring participants views of their social world. However, the objective of this work is vague as defined.	0	0	1	The researchers are using an ethographic approach to generatequalitative reseach. However the very nature of ethnographic reseach might make clear aims difficult to formulate prior to undertaking the reasearch and and a clear research question hasn't been stated . There is validity to their approach but will naturally contrast to the 'positivist science', ie empirical, replicable approach that quantative reserch would generate. In the abstract the authors
**2. Is a qualitative methodology appropriate?** Consider: (1) if the research seeks to interpret or illuminate the actions and / or subjective experiences of research participants; (2) is qualitative research the right methodology for addressing the research goal?	1	0	0	Clear description of the ethnographic approach, which aligns with the stated emphasis.	1	0	0	The ethnographic process is well descibed and is linked with quantitative and economic components
**3. Was the research design appropriate to address the aims of the research?** Consider: if the researcher has justified the research design (e.g. have they discussed how they decided which method to use)?	0	1	0	Aims are poorly defined which limits the ability to establish the appropriateness of the approach.	0	1	0	There are limitations to the descsriptions of the aims of the reaseach and why an ethnogrphaic approach was chosen specifically
**4. Was the recruitment strategy appropriate to the aims of the research?** Consider: (1) if the researcher has explained how the participants were selected; (2) if they explained why the participants they selected were the most appropriate to provide access to the type of knowledge sought by the study; (3) if there are any discussions around recruitment (e.g. why some people chose not to take part).	0	1	0	Approach to sampling, observation etc not well defined.	0	1	0	Since the aims are limited in their description it is hard to say whether the approach to sampling and recruitment was optimal or appropriate
**5. Was the data collected in a way that addressed the research issue?** Consider: (1) if the setting for data collection was justified; (2) if it is clear how data were collected (e.g. focus group, semi-structured interview etc); (3) if the researcher has justified the methods chosen; (4) if the researcher has made the methods explicit (e.g. for interview method, is there an indication of how the interviews were conducted, or did they use a topic guide); (5) if the methods were modified during the study. If so has the researcher explained how and why; (6) if the form of data is clear (e.g. tape recordings, video materials, notes); (7) if the researcher has discussed saturation of the data.	0	1	0	Methods align with the designated approach, however limited detail / depth is provided to substantiate findings.	0	1	0	Methods section provides a lot of detail. (Open-ended and semistructured interviews, history taking, review of written records. Field work was daily over 2.5 years), but the specific aims of each of these tasks were not clearly stated
**6. Has the relationship between the researcher and the participants been adequately considered?** Consider: (1) if the researcher critically examined their own role, potential bias and influence during formulation of the research questions an data collection (including sample selection and choice of location); (2) how the researhcer responds to events during the study and whether they considered the implications of any changes in the research design.	0	1	0	Limited description provided about the researcher, their role, the nature of their relationships and the assumptions they brought to the work.	0	0	1	There is not detail provided about the reasearchers, their roles and whether they had clincial involvments with the subjects of the reaseach. If there were relationships, this would affect the quality and bias the work.
**7. Have ethical issues been taken into consideration?** Consider: (1) is there sufficient details of how the research was explained to participants for the reader to assess whether ethical standards were maintained; (2) if the reseacher has discussed issues raised by the study (e.g. issues around informed consent or confidentiality or how they have handled the effects of the study on the participants during and after the study); (3) if approval has been sought from the ethics committee.	0	1	0	Not considered in the manuscript.	0	0	1	No mentions of ethics approval, no mention of any specific ethiical or boundary issues that were considered or whether participants had the opportunity to provide fully informed consent
**8. Was the data analysis sufficiently rigorous?** Consider: (1) if there is an in-depth description of the analysis process; (2) if thematic analysis is used. If so, is it clear how the categories/themes were derived from the data?; (3) whether the researcher explains how the data presented were selected from the original sample to demonstrate the analysis process; (4) if sufficent data are presented to support the findigns; (5) to what extent contradictory data are taken into account; (6) whether the researcher critically examined their own role, potential bias and influence during the analysis and selection of data for presentation.	0	1	0	The analytic process is inadequately describe, however supportive extracts are provided in the description and justification of findings.	0	0	1	There is insufficient evidence of a rigorous process around the analytic process. Thisraises questions about how the themes were generated. The results are presented in a table of 'common responses and observations' and further descriptions of results are in an unstructured narrative way
**9. Is there a clear statement of the findings?** Consider: (1) if the findings are explicit; (2) if there is adequate discussion of the evidence both for and against the researchers argument; (3) if the researcher has discussed the credibility of their findings (e.g. triangulation, respondent validation, more than one analyst); (4) if the findings are discussed in relation to the original research question.	0	1	0	Relationships between concepts were not well established. Content and descriptive focus.	0	1	0	The paper overall was descriptive and without a clear recent question and rigorous process of analysisg the data it would be difficult to justify their conclusions.
**10. How valuable is the research?** Consider: (1) if the researcher discusses the contribution the study makes to existing knowledge or understanding e.g. do they consider the findings in relation to current practice or policy, or relevant research-based literature?; (2) if they identify new areas where research is necessary; (3) if the researchers have discussed whether or how the findings can be transferred to other populations or considered ways the research may be used.	0	1	0	Implications for future practice not clearly communicated. Limitations as presented appear to emphasise the challenges faced in conducting ethnographic work, rather than consideration of the limitations in the methods and analytic process applied. The novelty of this work in the Australian context brings value, however limtiations in the description of the analytic process limits credibility.	0	1	0	Comprehensive research is necessary to establish this cost and clinical effectiveness of these services, and an ethnographic research approach appears to be essential for this as well as quantitative approach. This sresearch provides value to an underreasearched area, but the methodological flaws are limitations from which conclusions can be drawn.

**ADDITIONAL RATING: Quality rating**	**Initials**	**Rating**
Rater #1	SP	Poor
Rater #2	GH	Poor

Additional Comments (If LOW/POOR, please state why): There is limited description of the methods including sampling, observation methods, relationships between interviewer(s) and participants etc. This limits the trustworthiness of the findings reported.

CASP Qualitative Checklist

Study: **Parker S, Meurk C, Newman E, Fletcher C, Swinson I, Dark F. Understanding consumers' initial expectations of community-based residential mental health rehabilitation in the context of past experiences of care: A mixed-methods pragmatic grounded theory analysis. International journal of mental health nursing. 2018.**

	RATER 1				RATER 2			
Criteria	Yes	Can't tell	No	Justification	Yes	Can't tell	No	Justification
**1. Was there a clear statement of the aims of the research?** Consider: (1) what were the goals of the research; (2) why was it thought important; (3) its relevance.	1	0	0	"This study aims to overcome this gap by exploring consumers' expectations of community-based recovery oriented residential rehabilitation units, in comparison with their previous experiences of mental health care."<br><br>Aim clearly articulated and justified with reference to the pathway to residential rehabilitation care (across multiple service areas) and problems of engagement. Relevance of qualitative research also articulated.<br><br>"The interview schedule explored three topics: how participants came to be at the CCU, expectations of the CCU experience, and comparative expectations to previous experiences of mental health care (Parker et al. 2016b)....."<br><br>Interview schedule clearly described (with elaboration in published protocol) with clear questions of relevance to the topic focus.	1	0	0	"This study aims to overcome this gap by exploring con-sumers' expectations of community-based recovery- oriented residential rehabilitation units, in comparison with their previous experiences of mental health care' using semi-structured interviews."<br>Well contextualised in introduction.
**2. Is a qualitative methodology appropriate?** Consider: (1) if the research seeks to interpret or illuminate the actions and / or subjective experiences of research participants; (2) is qualitative research the right methodology for addressing the research goal?	1	0	0	Exploratory goal fits with the qualitative methods chosen.	1	0	0	Yes a qualitative method is appropriate in order to investigate the subjective experience of the CCU residents, in terms of their expectations of CCU and their previous experience of care.
**3. Was the research design appropriate to address the aims of the research?** Consider: if the researcher has justified the research design (e.g. have they discussed how they decided which method to use)?	1	0	0	Articulated in parent study protocol and justified in text.	1	0	0	There is detailed discussion of this in the article
**4. Was the recruitment strategy appropriate to the aims of the research?** Consider: (1) if the researcher has explained how the participants were selected; (2) if they explained why the participants they selected were the most appropriate to provide access to the type of knowledge sought by the study; (3) if there are any discussions around recruitment (e.g. why some people chose not to take part).	0	1	0	Convenience sampling approach. Did not use purposive sampling as would be typically employed in grounded theory. However, the approach was limited by the small potential sample pool. Iterative sampling to achieve saturation was used, however sampling was to thematic not theoretical saturation.	0	1	0	Convenience sample and not a purposive sample as would be normally used in grounded theory.
**5. Was the data collected in a way that addressed the research issue?** Consider: (1) if the setting for data collection was justified; (2) if it is clear how data were collected (e.g. focus group, semi-structured interview etc); (3) if the researcher has justified the methods chosen; (4) if the researcher has made the methods explicit (e.g. for interview method, is there an indication of how the interviews were conducted, or did they use a topic guide); (5) if the methods were modified during the study. If so has the researcher explained how and why; (6) if the form of data is clear (e.g. tape recordings, video materials, notes); (7) if the researcher has discussed saturation of the data.	1	0	0	Clear description provided in the study and parent study protocol of the data collection approach.	1	0	0	Clear description in the article and in the study protocol
**6. Has the relationship between the researcher and the participants been adequately considered?** Consider: (1) if the researcher critically examined their own role, potential bias and influence during formulation of the research questions an data collection (including sample selection and choice of location); (2) how the researhcer responds to events during the study and whether they considered the implications of any changes in the research design.	1	0	0	This is explicitly explored in the parent study protocol as well as the limitations. Issues relating to dual roles in the research team acknowledged and the potential impact considered in limitations.	1	0	0	The parent study protocol has detail about this
**7. Have ethical issues been taken into consideration?** Consider: (1) is there sufficient details of how the research was explained to participants for the reader to assess whether ethical standards were maintained; (2) if the reseacher has discussed issues raised by the study (e.g. issues around informed consent or confidentiality or how they have handled the effects of the study on the participants during and after the study); (3) if approval has been sought from the ethics committee.	0	1	0	Ethical approval is documented. Ethical issues considered in the protocol. Issues arising during the study not documented.	1	0	0	There is detail in the protocol in addition to the article, ethical approval was south by the relevant local committee.
**8. Was the data analysis sufficiently rigorous?** Consider: (1) if there is an in-depth description of the analysis process; (2) if thematic analysis is used. If so, is it clear how the categories/themes were derived from the data?; (3) whether the researcher explains how the data presented were selected from the original sample to demonstrate the analysis process; (4) if sufficent data are presented to support the findigns; (5) to what extent contradictory data are taken into account; (6) whether the researcher critically examined their own role, potential bias and influence during the analysis and selection of data for presentation.	1	0	0	Clear description is provided of the approach to the analysis. Multiple extracts provided in support of themes. Process of selection of illustrative extracts is documented. Some evidence of critical reflection on reflexivity. Use of respondent verification.	1	0	0	The data analysis is very rigorous and described in detail in the article.
**9. Is there a clear statement of the findings?** Consider: (1) if the findings are explicit; (2) if there is adequate discussion of the evidence both for and against the researchers argument; (3) if the researcher has discussed the credibility of their findings (e.g. triangulation, respondent validation, more than one analyst); (4) if the findings are discussed in relation to the original research question.	1	0	0	Findings clearly articulated and the relationship between themes is documented. Respondent validation was used and this supports the credibility.	1	0	0	There is clear description of the findings including a diagrammatical representation, and the themes are discussed in detail supported by evidence from the interviews
**10. How valuable is the research?** Consider: (1) if the researcher discusses the contribution the study makes to existing knowledge or understanding e.g. do they consider the findings in relation to current practice or policy, or relevant research-based literature?; (2) if they identify new areas where research is necessary; (3) if the researchers have discussed whether or how the findings can be transferred to other populations or considered ways the research may be used.	0	1	0	Provides information relating to an unresearched / under-researched topic. Case-to-case transferability issues raised in limitations. However, conclusion does draw out general prinicples of relevance to transfer and detailed description of context and participants facilitates informed decision from the reader about the relevance of this to other settings.	1	0	0	Since there is limited evidence in the literature, this article provides valuable insights about rehabilitation units and is methodologically rigorous. It particularly provides context to services in Queensland.

**ADDITIONAL RATING: Quality rating**	Initials	Rating
Rater #1	DS	Good
Rater #2	GH	Good

Additional Comments (If LOW/POOR, please state why): NA

CASP Qualitative Checklist

Study: **Parker S, Dark F, Newman E, Hanley D, McKinlay W, Meurk C. Consumers' understanding and expectations of a community-based recovery-oriented mental health rehabilitation unit: a pragmatic grounded theory analysis. Epidemiology and psychiatric sciences. 2017:1-10.**

	RATER 1				RATER 2			
**Criteria**	**Yes**	**Can't tell**	**No**	**Justification**	**Yes**	**Can't tell**	**No**	**Justification**
**1. Was there a clear statement of the aims of the research?** Consider: (1) what were the goals of the research; (2) why was it thought important; (3) its relevance.	1	0	0	"The present study aims to fill this gap by exploring the expectations consumers hold when they commence at a residential rehabilitation service for people affected by severe mentalillness in Australia called a Community Care Unit (CCU)..."<br>Exploratory aim well defined and contextualised. Justification of the relevance contextualised with reference to the known problem of engagement.<br>"The interview schedule was framed by three topics about the CCU: how participants came to be there; expectations of the experience; and expectations of how this would compare to previous mental health care experiences...."<br>Interview schedule clearly described (with elaboration in published protocol) with clear questions of relevance to the topic focus.	1	0	0	Aims are clearly stated in intro section- this study is exploring there difference between consumers expectations of care when the commence at a CCU and their experiences there, and justified as there is limited literature in this area. There is detail provided about the interview schedule used.
**2. Is a qualitative methodology appropriate?** Consider: (1) if the research seeks to interpret or illuminate the actions and / or subjective experiences of research participants; (2) is qualitative research the right methodology for addressing the research goal?	1	0	0	Exploratory goal fits with the qualitative methods chosen.	1	0	0	A qualitative rather than quantitative approach is appropriate as consumer opinions are being sought. The approach is suitable for the research question.
**3. Was the research design appropriate to address the aims of the research?** Consider: if the researcher has justified the research design (e.g. have they discussed how they decided which method to use)?	1	0	0	Articulated in parent study protocol and justified in text.	1	0	0	Yes the research design is appropriate for the research question
**4. Was the recruitment strategy appropriate to the aims of the research?** Consider: (1) if the researcher has explained how the participants were selected; (2) if they explained why the participants they selected were the most appropriate to provide access to the type of knowledge sought by the study; (3) if there are any discussions around recruitment (e.g. why some people chose not to take part).	0	1	0	Convenience sampling approach. Did not use purposive sampling as would be typically employed in grounded theory. However, the approach was limited by the small potential sample pool. Iterative sampling to achieve saturation was used, however sampling was to thematic not theoretical saturation.	0	1	0	The researchers have used a convenience sample of 48 patients recruited from 3 CCUs during the timeframe specified. There is no mention of why some chose not to participate.
**5. Was the data collected in a way that addressed the research issue?** Consider: (1) if the setting for data collection was justified; (2) if it is clear how data were collected (e.g. focus group, semi-structured interview etc); (3) if the researcher has justified the methods chosen; (4) if the researcher has made the methods explicit (e.g. for interview method, is there an indication of how the interviews were conducted, or did they use a topic guide); (5) if the methods were modified during the study. If so has the researcher explained how and why; (6) if the form of data is clear (e.g. tape recordings, video materials, notes); (7) if the researcher has discussed saturation of the data.	1	0	0	Clear description provided in the study and parent study protocol of the data collection approach.	1	0	0	The data collection methods are clearly described in the article
**6. Has the relationship between the researcher and the participants been adequately considered?** Consider: (1) if the researcher critically examined their own role, potential bias and influence during formulation of the research questions an data collection (including sample selection and choice of location); (2) how the researhcer responds to events during the study and whether they considered the implications of any changes in the research design.	1	0	0	This is explicitly explored in the parent study protocol as well as the limitations. Adaptation to interview schedule on the basis of initial interviews explained.	1	0	0	The involvement of researcher and their existing role in the CCUs is clearly discussed and how modifications were made in light of this to reduce bias.
**7. Have ethical issues been taken into consideration?** Consider: (1) is there sufficient details of how the research was explained to participants for the reader to assess whether ethical standards were maintained; (2) if the reseacher has discussed issues raised by the study (e.g. issues around informed consent or confidentiality or how they have handled the effects of the study on the participants during and after the study); (3) if approval has been sought from the ethics committee.	0	1	0	Ethical approval is documented. Ethical issues considered in the protocol. Issues arising during the study not documented.	1	0	0	Ethical approval was sought by the appropriate Metro South Committee.
**8. Was the data analysis sufficiently rigorous?** Consider: (1) if there is an in-depth description of the analysis process; (2) if thematic analysis is used. If so, is it clear how the categories/themes were derived from the data?; (3) whether the researcher explains how the data presented were selected from the original sample to demonstrate the analysis process; (4) if sufficent data are presented to support the findigns; (5) to what extent contradictory data are taken into account; (6) whether the researcher critically examined their own role, potential bias and influence during the analysis and selection of data for presentation.	1	0	0	Clear description is provided of the approach to the analysis. Multiple extracts provided in support of themes. Process of selection of illustrative extracts is documented. Some evidence of critical reflection on reflexivity.	1	0	0	There is extensive discussion of the data analysis. Direct quotations from interviews are used to support the themes generated.
**9. Is there a clear statement of the findings?** Consider: (1) if the findings are explicit; (2) if there is adequate discussion of the evidence both for and against the researchers argument; (3) if the researcher has discussed the credibility of their findings (e.g. triangulation, respondent validation, more than one analyst); (4) if the findings are discussed in relation to the original research question.	1	0	0	Findings clearly articulated and the relationship between themes is documented. Respondent validation was used.	1	0	0	Illustration of themes provided in addition to extensive discussion in the article.
**10. How valuable is the research?** Consider: (1) if the researcher discusses the contribution the study makes to existing knowledge or understanding e.g. do they consider the findings in relation to current practice or policy, or relevant research-based literature?; (2) if they identify new areas where research is necessary; (3) if the researchers have discussed whether or how the findings can be transferred to other populations or considered ways the research may be used.	0	1	0	Provides information relating to an unresearched / under-researched topic. Case-to-case transferability issues raised in limitations. However, conclusion does draw out general prinicples of relevance to transfer and detailed description of context and participants facilitates informed decision from the reader about the relevance of this to other settings.	1	0	0	This study contributes to an under researched area, particularly in Queensland context. Some limitations are discussed in the conclusion

**ADDITIONAL RATING: Quality rating**	**Initials**	**Rating**
Rater #1	DS	Good
Rater #2	GH	Good

Additional Comments (If LOW/POOR, please state why): NA

CASP Qualitative Checklist

Study: **Parker, S., et al. (2016). "Reality of working in a community-based, recovery-oriented mental health rehabilitation unit: A pragmatic grounded theory analysis." Int J Ment Health Nurs.**

	RATER 1				RATER 2			
**Criteria**	**Yes**	**Can't tell**	**No**	**Justification**	**Yes**	**Can't tell**	**No**	**Justification**
**1. Was there a clear statement of the aims of the research?** Consider: (1) what were the goals of the research; (2) why was it thought important; (3) its relevance.	1	0	0	Focus on exploring staff experience and understanding was made explicit.	1	0	0	There is a clear and explicit statement about the goals and relevance of this study and adequately contextualised
**2. Is a qualitative methodology appropriate?** Consider: (1) if the research seeks to interpret or illuminate the actions and / or subjective experiences of research participants; (2) is qualitative research the right methodology for addressing the research goal?	1	0	0	Exploratory goal fits with the qualitative methods chosen.	1	0	0	Rigorous methodological approach applied
**3. Was the research design appropriate to address the aims of the research?** Consider: if the researcher has justified the research design (e.g. have they discussed how they decided which method to use)?	1	0	0	Articulated in parent study protocol.	1	0	0	Justification of methods along with quality assurance measures detailed in original protocol and reference provided
**4. Was the recruitment strategy appropriate to the aims of the research?** Consider: (1) if the researcher has explained how the participants were selected; (2) if they explained why the participants they selected were the most appropriate to provide access to the type of knowledge sought by the study; (3) if there are any discussions around recruitment (e.g. why some people chose not to take part).	0	1	0	Did not use purposive sampling as would be typically employed in grounded theory. However, the approach was limited by the small potential sample pool. Iterative sampling to achieve saturation was used. Additionally, the consenting participants is nearly exhaustive of the potential pool.	1	0	0	Convenience sample of almost all available participants at the CCU and explanation of process of recruitment provided
**5. Was the data collected in a way that addressed the research issue?** Consider: (1) if the setting for data collection was justified; (2) if it is clear how data were collected (e.g. focus group, semi-structured interview etc); (3) if the researcher has justified the methods chosen; (4) if the researcher has made the methods explicit (e.g. for interview method, is there an indication of how the interviews were conducted, or did they use a topic guide); (5) if the methods were modified during the study. If so has the researcher explained how and why; (6) if the form of data is clear (e.g. tape recordings, video materials, notes); (7) if the researcher has discussed saturation of the data.	1	0	0	Clear description provided in the study and parent study protocol of the data collection approach.	1	0	0	A detailed protocol was published describing the data collection strategies and setting
**6. Has the relationship between the researcher and the participants been adequately considered?** Consider: (1) if the researcher critically examined their own role, potential bias and influence during formulation of the research questions an data collection (including sample selection and choice of location); (2) how the researhcer responds to events during the study and whether they considered the implications of any changes in the research design.	1	0	0	This is explicitly explored in the parent study protocol as well as the ,imitations.	1	0	0	Dual role of researcher outlined and author gave consideration of power differential as his role as a senior CCU senior staff member, along with the impact this could have on discouraging more negative views of CCU
**7. Have ethical issues been taken into consideration?** Consider: (1) is there sufficient details of how the research was explained to participants for the reader to assess whether ethical standards were maintained; (2) if the reseacher has discussed issues raised by the study (e.g. issues around informed consent or confidentiality or how they have handled the effects of the study on the participants during and after the study); (3) if approval has been sought from the ethics committee.	1	0	0	Ethical approval is documented. Ethical issues considered in the protocol. Issues arising during the study not documented.	1	0	0	Sufficient detail around ethical approval provided
**8. Was the data analysis sufficiently rigorous?** Consider: (1) if there is an in-depth description of the analysis process; (2) if thematic analysis is used. If so, is it clear how the categories/themes were derived from the data?; (3) whether the researcher explains how the data presented were selected from the original sample to demonstrate the analysis process; (4) if sufficent data are presented to support the findigns; (5) to what extent contradictory data are taken into account; (6) whether the researcher critically examined their own role, potential bias and influence during the analysis and selection of data for presentation.	1	0	0	Clear description is provided of the approach to the analysis. Multiple extracts provided in support of themes. Process of selection of illustrative extracts is documented. Some evidence of critical reflection on reflexivity.	1	0	0	There is indepth description of the analysis process, thematic analysis and description of themes that emerged.
**9. Is there a clear statement of the findings?** Consider: (1) if the findings are explicit; (2) if there is adequate discussion of the evidence both for and against the researchers argument; (3) if the researcher has discussed the credibility of their findings (e.g. triangulation, respondent validation, more than one analyst); (4) if the findings are discussed in relation to the original research question.	1	0	0	Findings clearly articulated and the relationship between themes is documented. Responded validation was used.	1	0	0	Explicit, clear explanation of the findings and conclusions drawn
**10. How valuable is the research?** Consider: (1) if the researcher discusses the contribution the study makes to existing knowledge or understanding e.g. do they consider the findings in relation to current practice or policy, or relevant research-based literature?; (2) if they identify new areas where research is necessary; (3) if the researchers have discussed whether or how the findings can be transferred to other populations or considered ways the research may be used.	0	1	0	Provides information relating to an unresearched / under-researched topic. Case-to-case transferability not explicitly discussed but conclusion does draw out general prinicples of relevance to transfer.	1	0	0	This is a methodologically rigorous study that makes a contribution to an under researched area

**ADDITIONAL RATING: Quality rating**	**Initials**	**Rating**
Rater #1	DS	Good
Rater #2	GH	Good

Additional Comments (If LOW/POOR, please state why): NA

NIHL Checklist - Quality Assessment Tool for Case Series Studies

Study:

Smith, G, Williams, T and Lefay, L. (2009). Evaluating the Hawthorn House Rehabilitation Service. Department of Health, Perth.
(http://www.health.wa.gov.au/mhpr/docs/Evaluating_Hawthorn_House_Rehabilitation_Service2008.pdf)

	RATER 1				RATER 2			
**Criteria**	**Yes**	**No**	**Other (CD, NR, NA)***	**Justification**	**Yes**	**No**	**Other (CD, NR, NA)***	**Justification**
1. Was the study question or objective clearly stated?	1	0		Intention to evaluation HH model clearly stated but the approach does not justify the 'unequivocal' conclusions	1	0	0	Report has clearly stated brief, with four main objectives
2. Was the study population clearly and fully described, including a case definition?	0	0	CD	Use of local terminology impacts the interpretation	0	0	CD	This is unclear
3. Were the cases consecutive?	0	0	CD	Inadequate description of sampling, assumption is complete data	0	0	CD	As above
4. Were the subjects comparable?	0	0	CD	Nil analysis	0	0	CD	Insufficient detail
5. Was the intervention clearly described?	0	1		Service model described but with inadequate detail	1	0	0	There is a description of Hawthorn House
6. Were the outcome measures clearly defined, valid, reliable, and implemented consistently across all study participants?	0	0	NA	Nil analysis	0	1	0	N/A
7. Was the length of follow-up adequate?	0	1		Nil analysis	0	1	0	N/A
8. Were the statistical methods well-described?	0	0	NA	Nil analysis	0	1	0	N/A
9. Were the results well-described?	0	1		Inadeqate description	0	1	0	N/A

**Quality rating**	**Initials**	**Rating**
Rater #1	SP	Poor
Rater #2	GH	Poor

Additional Comments (If LOW/POOR, please state why): Conclusions drawn are not adequately supported by the data, minimal description of methodology

* CD, cannot determine; NA, not applicable; NR, not reported

CASP Qualitative Checklist

Study: **Smith, G, Williams, T and Lefay, L. (2009). Evaluating the Hawthorn House Rehabilitation Service. Department of Health, Perth. (http://www.health.wa.gov.au/mhpr/docs/Evaluating_Hawthorn_House_Rehabilitation_Service2008.pdf)**

	RATER 1				RATER 2			
**Criteria**	**Yes**	**Can't tell**	**No**	**Justification**	**Yes**	**Can't tell**	**No**	**Justification**
**1. Was there a clear statement of the aims of the research?** Consider: (1) what were the goals of the research; (2) why was it thought important; (3) its relevance.	1	0	0	Goal well described	1	0	0	Report has clearly stated brief, with four main objectives
**2. Is a qualitative methodology appropriate?** Consider: (1) if the research seeks to interpret or illuminate the actions and / or subjective experiences of research participants; (2) is qualitative research the right methodology for addressing the research goal?	0	1	0	Nil description of methodology	0	1	0	The methodology hasn't been well described
**3. Was the research design appropriate to address the aims of the research?** Consider: if the researcher has justified the research design (e.g. have they discussed how they decided which method to use)?	0	1	0	Nil description of methodology	0	1	0	Unable to comment as little detail provided
**4. Was the recruitment strategy appropriate to the aims of the research?** Consider: (1) if the researcher has explained how the participants were selected; (2) if they explained why the participants they selected were the most appropriate to provide access to the type of knowledge sought by the study; (3) if there are any discussions around recruitment (e.g. why some people chose not to take part).	0	1	0	Nil description of methodology	0	1	0	As above
**5. Was the data collected in a way that addressed the research issue?** Consider: (1) if the setting for data collection was justified; (2) if it is clear how data were collected (e.g. focus group, semi-structured interview etc); (3) if the researcher has justified the methods chosen; (4) if the researcher has made the methods explicit (e.g. for interview method, is there an indication of how the interviews were conducted, or did they use a topic guide); (5) if the methods were modified during the study. If so has the researcher explained how and why; (6) if the form of data is clear (e.g. tape recordings, video materials, notes); (7) if the researcher has discussed saturation of the data.	0	1	0	Nil description of methodology	0	1	0	As above
**6. Has the relationship between the researcher and the participants been adequately considered?** Consider: (1) if the researcher critically examined their own role, potential bias and influence during formulation of the research questions an data collection (including sample selection and choice of location); (2) how the researhcer responds to events during the study and whether they considered the implications of any changes in the research design.	0	0	1	Nil description of methodology	0	1	0	As above
**7. Have ethical issues been taken into consideration?** Consider: (1) is there sufficient details of how the research was explained to participants for the reader to assess whether ethical standards were maintained; (2) if the reseacher has discussed issues raised by the study (e.g. issues around informed consent or confidentiality or how they have handled the effects of the study on the participants during and after the study); (3) if approval has been sought from the ethics committee.	0	1	0	Nil description of methodology	0	1	0	As above
**8. Was the data analysis sufficiently rigorous?** Consider: (1) if there is an in-depth description of the analysis process; (2) if thematic analysis is used. If so, is it clear how the categories/themes were derived from the data?; (3) whether the researcher explains how the data presented were selected from the original sample to demonstrate the analysis process; (4) if sufficent data are presented to support the findigns; (5) to what extent contradictory data are taken into account; (6) whether the researcher critically examined their own role, potential bias and influence during the analysis and selection of data for presentation.	0	1	0	Nil description of methodology	0	1	0	As above
**9. Is there a clear statement of the findings?** Consider: (1) if the findings are explicit; (2) if there is adequate discussion of the evidence both for and against the researchers argument; (3) if the researcher has discussed the credibility of their findings (e.g. triangulation, respondent validation, more than one analyst); (4) if the findings are discussed in relation to the original research question.	1	0	0	Yes but in the absence of description of methodology these are not trustworthy	0	1	0	As above
**10. How valuable is the research?** Consider: (1) if the researcher discusses the contribution the study makes to existing knowledge or understanding e.g. do they consider the findings in relation to current practice or policy, or relevant research-based literature?; (2) if they identify new areas where research is necessary; (3) if the researchers have discussed whether or how the findings can be transferred to other populations or considered ways the research may be used.	0	1	0	Lack of trustworthiness limits value.	0	1	0	As above

**ADDITIONAL RATING: Quality rating**	**Initials**	**Rating**
Rater #1	SP	Poor
Rater #2	GH	Poor

Additional Comments (If LOW/POOR, please state why): No description of the methodology is provided, this undermines the trustworthiness of the findings.

NIHL Checklist - Quality Assessment Tool for Case Series Studies

Study: **Trauer, T. (2001). "Symptom severity and personal functioning among patients with schizophrenia discharged from long-term hospital care into the community." Community Ment Health J 37(2): 145-155.**

	RATER 1				RATER 2			
**Criteria**	**Yes**	**No**	**Other (CD, NR, NA)***	**Justification**	**Yes**	**No**	**Other (CD, NR, NA)***	**Justification**
1. Was the study question or objective clearly stated?	0	1		Exploratory focus. No specific hypotheses were stated, however the background contextualised the study with reference to the failure to observe symptomatic change in an equivalent UK setting 6/12 post transition.	0	1		Objective was to measure symptoms and functioning of residents and explore variations after one year using PANSS and MCAS. I don't think the study question is clearly defined, ?did they anticipate an improvement or disimprovement after one year. We also don't know how long some of the residents had been out of hospital already so they were perhaps at different stages of transition (the purpose if the study is to examine the transition from institution to community)
2. Was the study population clearly and fully described, including a case definition?	1	0		Full sampling of all 20 residents present at a single CCU. Model of service operant was clearly described. Adequate description of demographics including age, illness duration, diagnosis and co-morbidity, mental health act status, and financial situation.	1	0		Pragmatic sample from one CCU (18/20 residents) with demographic details, and other relevant information provided
3. Were the cases consecutive?	0	0	N/A	Comprehensive sampling of a single group.	0	1		Pragmatic sample from the CCU, not consecutive
4. Were the subjects comparable?	1	0		All resided at the same CCU, therefore the service level intervention was equivalent. Diagnostically homogenous. No consideration of how individual level confounders may have impacted the pattern of results.	1	0		All had psychotic dosorder, similar MHA status, co-morbidities, all on antipsychotic medication and resident at the CCU
5. Was the intervention clearly described?	1	0		Adequate description of CCU model provided.	1	0		There is a description of CCU provided in introduction. 2 interviewers giving PANSS and MCAS at baseline, then one interviewer at one year
6. Were the outcome measures clearly defined, valid, reliable, and implemented consistently across all study participants?	1	0		PANSS is well validated. MCAS is less widely use, but has been used to demonstrate functional change across several studies. Concerns have been raised about reliability and fit of the original factor structure (see Bassani et al, 2009). PANSS interviews were completed by 'trained' (do elaboration) staff in pairs (with known	1	0		PANSS and MCAS. These are valid tools. 2 interviewers, then 1 at one year. Process for Inter-rater reliability given in detail
7. Was the length of follow-up adequate?	1	0		1-year is an appropriate timeframe given studies in similar services in the UK have also failed to demonstrate symptomatic gains over a similar timeframe. However, further longitudinal follow-up would have given greater	1	0		Yes, after one year of residential rehab one would expect some clinical changes reflected in the scores
8. Were the statistical methods well-described?	1	0		Adequate description provided.	1	0		There is a lot of description of the statistical methods relating to inter-rater reliability, intercorrelations between scales, correlation of scores one year apart
9. Were the results well-described?	1	0		Adequate description provided.	1	0		There is detail provided about results i.e. symptom levels and functioning levels being positively related, broadly little changes in symptoms and functioning after one year and reference to similar findings in other studies

**Quality rating**	**Initials**	**Rating**
Rater #1	SP	Good
Rater #2	GH	Good

Additional Comments (If LOW/POOR, please state why): N/A

* CD, cannot determine; NA, not applicable; NR, not reported

NIHL Checklist - Quality Assessment Tool for Before-After (Pre-Post) Studies with no control group

Study: **Trauer T, Farhall J, Newton R, Cheung P. From long-stay psychiatric hospital to Community Care Unit: evaluation at 1 year. Social psychiatry and psychiatric epidemiology. 2001;36(8):416-9**

**Criteria**	**RATER 1** Yes	No	Other (CD, NR, NA)*	Justification	**RATER 2** Yes	No	Other (CD, NR, NA)*	Justification
1. Was the study question or objective clearly stated?	0	1		Objectives are not clearly stated.	0	1		They haven't stated a coherent objective or question, just an intention to measure various measures in the 1 year period of adjustment from hospital to CCU. There is no specific hypothesis
2. Were eligibility/selection criteria for the study population prespecified and clearly described?	0	0	CD	Aim of exhaustive sampling stated. No detail provided about the process that determined eligibility for consideration for CCU based care though - this may have been informative in understanding who was excluded / included in the cohort.	0	1		Very vague - 'all patients nominated for a CCU' who were long term hospitalised patients prior to this. No referral criteria for CCU specified, however detail about demographics, diagnosis, chronicity of patients is provided
3. Were the participants in the study representative of those who would be eligible for the test/service/intervention in the general or clinical population of interest?	1	0		Exhaustive sampling attempted.	1	0		There is detail provided in 'The patients' section about the participants in the study - their diagnosis, level of chronicity, medication, number of hospitalisations
4. Were all eligible participants that met the prespecified entry criteria enrolled?	1			See 3.	1	0		'125 patients in long term open wards and a few from elsewhere in the hospital with similar needs, were proposed for transfer to CCUs'
5. Was the sample size sufficiently large to provide confidence in the findings?	0	0	CD	No documentation provided in relation to power. In terms of describing the cohort the sample is adequate in that it	0	0	CD	Adequate sample but no reference to power calculations
6. Was the test/service/intervention clearly described and delivered consistently across the study population?	0	0	CD	Adequate description is provided with regards to the service model, but the extent to which this was realised consistently across sites is not documented.	0	0	CD	There were 5 different CCUs and 'interim' CCUs in this study, where the service delivery may differ and be different in ethos, staffing (for example clinical or peer work staff) which would affect the quality and consistency of interventions provided
7. Were the outcome measures prespecified, clearly defined, valid, reliable, and assessed consistently across all study participants?	0	1		There were high and variable levels of missing data across the measures. Limited information is provided to assist in understanding the mechanisms for this. The nature of the specifically developed carer and relatives quetionairre is not adequately described.	0	1		The objectives were to administer rating tools at 1 month pre move, 1 month post move, and at one year. The results are inconsistently reported in the article and no rationale of why data was incomplete.
8. Were the people assessing the outcomes blinded to the participants' exposures/interventions?	0	0	NR	Not documented	0	0	NR	Not stated
9. Was the loss to follow-up after baseline 20% or less? Were those lost to follow-up accounted for in the analysis?	0	0	NA		0	1		Yes lots of dropouts and missing data
10. Did the statistical methods examine changes in outcome measures from before to after the intervention? Were statistical tests done that provided p values for the pre-to-post changes?	0	0	CD	Pre and post assessments were completed (with missing data). P-values only reported for significant changes. No documentation in relation to correction for multiple comparison. No a priori specification of primary and secondary outcome foci. Several outcomes are only analysed at the descriptive level. Why is the PAQ only presented descriptively?	0	0	CD	PANSS, LQOLP, SOAS were administered pre and post move. All results, and particularly the tests of statistical significance i.e. P values were inconsistently and selectively reported (i.e. for SNA)
11. Were outcome measures of interest taken multiple times before the intervention and multiple times after the intervention (i.e., did they use an interrupted time-series design)?	0	1		No.	0	1		The rating tools (PANSS, LQOLP, SNA) were taken pre move and one year post move, SOAS administered pre. One month post, and one year post.
12. If the intervention was conducted at a group level (e.g., a whole hospital, a community, etc.) did the statistical analysis take into account the use of individual-level data to determine effects at the group level?	0	1		No documentation to suggest adjustment for individual level confounds, nor consideration of site level confounds.	0	1		While there is some background and demographic detail given about the participants, there is limited reference to patients individual confounders and their potential impact

**Quality rating**	Initials	Rating
Rater #1	SP	Poor
Rater #2	GH	Poor

Additional Comments (If POOR, please state why): Note that authors provide appropriate acknowledgement of the limitations of the naturalistic approach re missing data, absence of control. There are issues with data collection and completeness, inconsistent reporting of results and a lack of clear objectives.

* CD, cannot determine; NA, not applicable; NR, not reported
