# Supplementary material for: A systematic review of service models and evidence relating to the clinically operated community-based residential mental health rehabilitation for adults with severe and persisting mental illness in Australia
Source: BMC Psychiatry. 2019 Feb 4;19:55. doi: 10.1186/s12888-019-2019-5 (PMC6360669; doi:10.1186/s12888-019-2019-5)
Supplement: Supplementary file 3 — Service types and models. (PDF 99 kb) [file 12888_2019_2019_MOESM3_ESM.pdf]

Appendix 2: Detailed description of types & models of community-based & clinically operated residential rehabilitation for people affected by schizophrenia & related disorders in Australia.

Service type	Focus / objective	Service model	Timeframe	State	Physical environment	Philosophy of Care	Treatment and support	Source
Community based residential care	▪ Deinstitutionalisation of people with SPMI<br>▪ Permanent residence<br>▪ Accommodation, rehabilitation and support	Community Residences	1994-early 2000s	NSW	▪ Group homes (3x 10-bed), with single or twin share bedrooms and shared kitchen and dining facilities, OR apartment block (x1) with four self-contained units.<br>▪ Provision of indoor and outdoor recreation areas.<br>▪ Walking distance to shops and public transport.	▪ Intensive case management through an adapted model of Assertive Community Treatment<br>▪ Normalisation through treatment in a residential setting<br>▪ Rehabilitation focus	▪ 24-hour Multi-Disciplinary Team (MDT) support initially, with a step-wise reduction over time<br>▪ Individualised programs: living skills, planning for more independent living (e.g. budgeting, medication self-administration, vocational engagement)<br>▪ Prompting, modelling, task analysis, practical assistance and education<br>▪ Case managers facilitate access to external resources in the community.	[1-3]
		Community Care Unit	1996-early 2000s	VIC	▪ Purpose build cluster-style housing with up to 20-beds, providing a 'home-like environment' built close to shops and public transport.<br>▪ Provision of outdoor recreation and quiet spaces, as well as communal rooms for group activities, described<br>▪ Early iterations provided individual bedrooms and shared bathroom, kitchen and living areas to small groups of residents.<br>▪ Later iterations described mixed residential configurations including self-contained single-occupancy, dual occupancy and shared units	▪ The application of case management to a residential psychosocial rehabilitation program integrating clinical care and disability support<br>▪ Emphasis on accessing and utilising community-based services and resources	▪ 24-hour nursing supervision available<br>▪ Primary focus of 'living skills therapy' and medication adherence/monitoring<br>▪ Absence of group-based rehabilitation activities emphasised<br>▪ Behaviour therapy as the only documented individual therapy	[4-10]
Transitional residential rehabilitation	▪ Rehabilitation<br>▪ Focus on SPMI<br>▪ Transitional residential support		Early 2000s+	VIC QLD		▪ Transitional support over 6-24 months, but with acknowledgement that some consumers may require further extended care<br>▪ Recovery-oriented<br>▪ Strengths-based<br>▪ Rehabilitation focussed<br>▪ Individualised care planning	▪ 24-hour clinical staff support availability<br>▪ Individual therapeutic focus including: CBT; living skills development; structured leisure and physical activities; and evidence-based therapeutic groups.<br>▪ Provision of psychoeducation and support to carers, and promoting continued links between consumers and their carers<br>▪ Not authorised mental health services	[4, 11-16]
			2014+	QLD			▪ As above but with the additional availability of an integrated staffing configuration where Peer Support Workers occupy the majority of roles.	[11, 12, 17]
		Hawthorne House	2006-2009	WA	▪ Stand-alone 16-bed converted community hospital	▪ Psychiatric rehabilitation<br>▪ Commitment to recovery<br>▪ Biopsychosocial approach	▪ Maximum of 16 weeks of active structured psychiatric rehabilitation focussed on transitioning 'rehabilitation ready' hospitalised inpatients<br>▪ MDT (Occupational Therapy, Nursing, Social Work, Art therapy, Medical)	[18]
		Community Rehabilitation Centre	2007+	SA	▪ Three sites including 2 purpose-built (outer-urban location) and one re-purposed building (inner-urban location).<br>▪ Shared and individual living units are available, each with individual bedrooms.	▪ Recovery-oriented rehabilitation, including focus on prevention, early intervention, and voluntary participation<br>▪ Emphasis on viewing the person in the context of their family and significant others.	▪ 24-hour staff support, with staffing configuration including clinical and non-clinical (including peer) specialists<br>▪ Working in partnership with the community mental health team.<br>▪ Active and goal focused rehabilitation program including therapeutic interventions, skills development, building/rebuilding personal networks, and fostering community links<br>▪ Average residence <6 months<br>▪ Not Approved Treatment Centres	[19-21]
		Community Recovery Program	2014+	VIC	▪ 22-bed purpose-built unit, standalone co-located with a hospital service	▪ Recovery-focused rehabilitation<br>▪ working within a philosophy of 'home' and 'community', recovery and social inclusion, and consumer participation.<br>▪ Emphasis also on consumer-centred care, and family/carer involvement.	▪ Partnership model with clinical care provided by nursing staff employed by the health service and 'community health practitioners' with expertise in recovery-oriented psychosocial rehabilitation employed by a non-government organisation. Peer and family/care workers are included in staffing profile.<br>▪ Focus on living skills development, psychoeducation, recreation and fostering links with the community.<br>▪ Group programs are available.	[22-24]

REFERENCES:

1. Hobbs C, Newton L, Tennant C, Rosen A, Tribe K: **Deinstitutionalization for long-term mental illness: a 6-year evaluation**. *The Australian and New Zealand journal of psychiatry* 2002, **36**(1):60-66.
2. Newton L, Rosen A, Tennant C, Hobbs C, Lapsley HM, Tribe K: **Deinstitutionalisation for long-term mental illness: an ethnographic study**. *The Australian and New Zealand journal of psychiatry* 2000, **34**(3):484-490.
3. Hobbs C, Tennant C, Rosen A, Newton L, Lapsley HM, Tribe K, Brown JE: **Deinstitutionalisation for long-term mental illness: a 2-year clinical evaluation**. *The Australian and New Zealand journal of psychiatry* 2000, **34**(3):476-483.
4. Chopra P, Herrman HE: **The long-term outcomes and unmet needs of a cohort of former long-stay patients in Melbourne, Australia**. *Community mental health journal* 2011, **47**(5):531-541.
5. Hamden A, Newton R, McCauley-Elsom K, Cross W: **Is deinstitutionalization working in our community?** *International journal of mental health nursing* 2011, **20**(4):274-283.
6. Farhall J, Trauer T, Newton R, Cheung P: **Minimizing adverse effects on patients of involuntary relocation from long-stay wards to community residences**. *Psychiatric services (Washington, DC)* 2003, **54**(7):1022-1027.
7. Trauer T, Farhall J, Newton R, Cheung P: **From long-stay psychiatric hospital to Community Care Unit: evaluation at 1 year**. *Social psychiatry and psychiatric epidemiology* 2001, **36**(8):416-419.
8. Trauer T: **Symptom severity and personal functioning among patients with schizophrenia discharged from long-term hospital care into the community**. *Community mental health journal* 2001, **37**(2):145-155.
9. Farhall J, Trauer T, Newton R, Cheung P: **Community Care Units Evaluation Project: One year report. Melbourne: Victorian Government.** In.; 1999.
10. Gerrand V, Bloch S, Smith J, Goding M, Castle D: **Reforming mental health care in Victoria: a decade later**. *Australasian psychiatry : bulletin of Royal Australian and New Zealand College of Psychiatrists* 2007, **15**(3):181-184.
11. Parker S, Meurk C, Newman E, Fletcher C, Swinson I, Dark F: **Understanding consumers' initial expectations of community-based residential mental health rehabilitation in the context of past experiences of care: A mixed-methods pragmatic grounded theory analysis**. *International journal of mental health nursing* 2018.
12. Parker S, Dark F, Newman E, Hanley D, McKinlay W, Meurk C: **Consumers' understanding and expectations of a community-based recovery-oriented mental health rehabilitation unit: a pragmatic grounded theory analysis**. *Epidemiology and psychiatric sciences* 2017:1-10.
13. Parker S, Dark F, Newman E, Korman N, Rasmussen Z, Meurk C: **Reality of working in a community-based, recovery-oriented mental health rehabilitation unit: A pragmatic grounded theory analysis**. *International journal of mental health nursing* 2017, **26**(4):355-365.
14. Meehan T, Stedman T, Parker S, Curtis B, Jones D: **Comparing clinical and demographic characteristics of people with mental illness in hospital- and community-based residential rehabilitation units in Queensland**. *Aust Health Rev* 2017, **41**(2):139-143.
15. McKenna B, Oakes J, Fourniotis N, Toomey N, Furness T: **Recovery-Oriented Mental Health Practice in a Community Care Unit: An Exploratory Study**. *Journal of forensic nursing* 2016, **12**(4):167-175.
16. Department of Human Services: **Community Care (CCU) and secure extended care units (SECU). Program management circular.** In. Edited by Division MHBMHaACS. Victoria: Victorian Government; 2007.
17. Parker S, Dark F, Vilic G, Doyle C, Lendich B, O'Sullivan R, McCann K: **Integrated staffing model for mental health rehabilitation (in press)**. *Mental Health and Social Inclusion* 2016, **20**(2).
18. Smith G, Williams T, Lefay L: **Evaluating the Hawthorn House Rehabilitation Service**. In. Perth: Department of Health; 2009.
19. Barnett K, Guiver N, Cheok F: **Evaluation of the Three Community Rehabilitation Centres: FINAL REPORT**. In. Edited by Health S. South Australia: SA Health; 2011.
20. SA HEALTH MHU: **Community Mental Health Services Service Model: Community Recovery Centres**. In. South Australia; 2010.
21. Department of Health GoSA: **Mental Health Service: Community Rehabilitation Centres (CRCs)**. In. Edited by Department of Health GoSA. South Australia: Department of Health, Government of South Australia; 2010.
22. Saraf S, Newton R: **Care or recovery? Redefining residential rehabilitation**. *Australasian psychiatry : bulletin of Royal Australian and New Zealand College of Psychiatrists* 2017, **25**(2):161-163.
23. Wooldridge M: **Media release: New mental health recovery program opens at Austin Health**. In. Edited by Victorian Government. Victoria; 2014.
24. **Community Recovery Program** [http://www.austin.org.au/mentalhealth/NEAMHS/CRP/]
