# Supplementary material for: A systematic review of service models and evidence relating to the clinically operated community-based residential mental health rehabilitation for adults with severe and persisting mental illness in Australia
Source: BMC Psychiatry. 2019 Feb 4;19:55. doi: 10.1186/s12888-019-2019-5 (PMC6360669; doi:10.1186/s12888-019-2019-5)
Supplement: Supplementary file 4 — Consumer characteristics. (PDF 457 kb) [file 12888_2019_2019_MOESM4_ESM.pdf]

Appendix 3: Consumer characteristics

Source(s)		Service classification		Cohort details					Demographics						Restrictive Practice		Dx	Co-morbidity					Impairment		
Study	Reference(s)	Service Type	Service name	Cohort type	Sample size	State	Year START	Year END	Mean Age	Male	Australian born	ATSI[j]	Unemployment	Community admission	Involuntary treatment	Guardianship order	Primary Dx - F20-F29[k]	Substance use[l]	Developmental Disorder	Personality disorder	Acquired brain injury	Physical illness	Mean Total LSP-16	Mean total HoNOS	Mean CPZ eq[m] (mg)
**Included records presenting unique descriptive information about consumers[a,b]**																									
QMHBU[c] (2017)[d]	[1]	TRR +/-	CCU	Cross sectional (current residents)	241	QLD	2017	-	36	71%	86%	12%	70%	46%	58%	37%	85%	30%	-	-	-	53%	-	-	573.2
QMHBU (2015)[d]	[2]	TRR +/-	CCU	Cross sectional (current residents)	155	QLD	2015	-	37	76%	81%	8%	-	46%	65%	38%	90%	21%	5%	6%	-	50%	-	-	610.2
Parker et al. (2017, 2018)[d,e]	[3]	TRR +/-	CCU	Service commencement	24	QLD	2014	2016	30 (7.8)	75%	-	-	100%	67%	50%	-	87%	54%	8%	8%	4%	-	13 (7.5)	12 (6.4)	646 (411)
Meehan (2017) et al	[4]	TRR	CCU	Cross sectional (current residents)	115	QLD	2013	-	39	-	-	-	-	-	38%	36%	84%	-	-	-	-	44.30%	17.5 (8.7)	12.7 (6.4)	559 (314)
QMHBU (2011)	[5]	TRR	CCU	Cross sectional (current residents)	85	QLD	2011	-	38	69%	82%	8%	-	-	72%	33%	93%	27%	-	-	-	60%	-	-	684.7
QMHBU (2009)	[6]	TRR	CCU	Cross sectional (current residents)	85	QLD	2009	-	35	80%	88%	12%	-	-	67%	72%	94%	31%	-	-	-	29%	-	-	727.9
Barnett et al (2011)[f]	[7]	TRR	CRU	Case series	238	SA	2007	2010	32	75%	-	5%	-	81%[g]	7%	-	80%	4%	2%	4%	-	3%	-	16.5	-
QMHBU (2007)	[8]	TRR	CCU	Cross sectional (current residents)	92	QLD	2007	-	36	75%	89%	16%	-	-	67%	46%	93%	22%	-	-	-	44%	-	-	-
Smith, Williams & Lefay (2009)	[9]	TRR	Hawthorne House	Case series	39	WA	2006	2008	33	49%	-	-	-	0%[i]	-	-	71%	-	-	-	-	-	-	-	-
QMHBU (2005)	[10]	TRR	CCU	Cross sectional (current residents)	76	QLD	2005	-	35	67%	-	7%	-	-	70%	-	88%	-	-	-	-	22%	-	-	-
Trauer (2001)[h]	[11]	C-BRC	CCU	Case series	20	VIC	1997	1998	43 (9.8)	60%	-	-	-	0%	95%	-	100%	15%	15%	-	-	20%	-	-	936
Trauer (2001) et al[h]	[12]	C-BRC	CCU	Service commencement	125	VIC	1996	-	44	62%	-	-	-	0%	86%	-	97%	-	-	-	-	-	-	-	775
Hamden (2011) et al	[13]	C-BRC	CCU	Current and past service users	121	VIC	1996	2007	38	67%	-	-	-	-	-	-	-	-	-	-	-	-	-	-	-
Hobbs et al (2002)~	[14]	C-BRC	Community Residences	Service commencement	47	NSW	1994	-	41	53%	83%	-	-	0%	-	-	98%	-	2%	-	-	-	-	-	1127
Farhall et al. (2003)[h]	[15]	C-BRC	CCU	Service commencement	85	VIC	1994	1999	42 (9.8)	64%	-	-	-	0%	84%	-	92%	-	-	-	-	-	-	-	-
**Excluded records: Follow-up details only**																									
Chopra (2011) et al.	[16]	TRR	CCU	Follow-up (8 years post)	18	VIC	2003	-	-	-	-	-	100%	0%	-	-	100%	17%	28%	6%	-	83%	22.1	16.2	-
**Excluded records: Nil descriptive data available**																									
Munro (2007) et al	[17]	TRR	CCU	Single case study	1	-	2007	-	-	-	-	-	-	-	-	-	-	-	-	-	-	-	-	-	-
McKenna (2016) et al	[18]	TRR	CCU	Qualitative interviews	7	-	2014	-	-	-	-	-	-	-	-	-	-	-	-	-	-	-	-	-	-

Notes

a For longitudinal cohort data, the earliest time-point of data collection is added when available to each cell

b Where relevant and available standard deviations are listed in brackets ( ) following the mean score, where decimal points for the mean values are available these are presented at the level of one decimal point.

c Queensland Mental Health Benchmarking Unit (QMHBU) associated publications (formerly Service and Evaluation Research Unit)

d At least partial overlap is anticipated across these studies due to the combination of cross-sectional data and service commencement cohorts

e The same cohort is considered across both studies, only the reference for the earliest published study is provided

f The actual sample size for each variable is not consistent, n=238 represented maximal cohort size

g Referral data based on the total referrals during the study period (n=266) not the accepted referrals (n=238)

h Anticipated at least partial overlap of cohort data, as all records related to the initially deinstitutionalised Victorian consumer group

i Inferred from the documented requirement for service entry for referrals to be made from current inpatient mental health consumers

j Identification as being of Aboriginal and/or Torres Strait Islander descent

k Schizophrenia spectrum disorders reflecting diagnoses (or equivalent diagnoses) of the International Classification of Diseases codes F20-29.x

l Any co-morbid substance use disorder excluding tobacco-related disorders.

m Mean Chlorpromazine Dose equivalence (as documented in the associated record).

REFERENCES

1. Queensland Mental Health Benchmarking Unit (QMHBU): **Community Care Units (CCU) Benchmarking Report 2017**. In.; 2017.
2. Queensland Mental Health Benchmarking Unit: **Community Care Units: Benchmarking report 2015 - Queensland Mental Health Benchmarking Unit**. In. Wacol, Qld: Mental Health, Alcohol and Other Drugs Branch, Department of Health (Queensland); 2015.
3. Parker S, Dark F, Newman E, Hanley D, McKinlay W, Meurk C: **Consumers' understanding and expectations of a community-based recovery-oriented mental health rehabilitation unit: a pragmatic grounded theory analysis**. *Epidemiology and psychiatric sciences* 2017:1-10.
4. Meehan T, Stedman T, Parker S, Curtis B, Jones D: **Comparing clinical and demographic characteristics of people with mental illness in hospital- and community-based residential rehabilitation units in Queensland**. *Aust Health Rev* 2017, **41**(2):139-143.
5. Queensland Mental Health Benchmarking Unit: **Multi-site benchmarking of Community Care Units and Extended Treatment & Rehabilitation Mental Health Services - Comparative benchmarking report 2011 - Queensland Mental Health Benchmarking Unit**. In. Wacol, Queensland: Queensland Mental Health Benchmarking Unit,; 2011.
6. Jones D, Neuendorf K, Denkel N: **Multi-site benchmarking of Extended Treatment & Rehabilitation and Dual Diagnosis Inpatient Mental Health Services**. In. Wacol, Queensland: Queensland Mental Health Benchmarking Unit; 2009.
7. Barnett K, Guiver N, Cheok F: **Evaluation of the Three Community Rehabilitation Centres: FINAL REPORT**. In. Edited by Health S. South Australia: SA Health; 2011.
8. Davidson F, Jones D, Neuendorf K: **Multi-site benchmarking of Extended Treatment & Rehabilitation and Dual Diagnosis Inpatient Mental Health Services - Benchmarking report (2007)**. In. Wacol, Queensland: Queensland Mental Health Benchmarking Unit; 2007.
9. Smith G, Williams T, Lefay L: **Evaluating the Hawthorn House Rehabilitation Service**. In. Perth: Department of Health; 2009.
10. Meehan T, Neuendorf K: **Multi-site benchmarking of Extended Treatment & Rehabilitation and Dual Diagnosis Services - Benchmarking Report (2005)**. In. Wacol, Queensland: Service Evaluation and Research Unit, The Park Centre for Mental Health, Brisbane; 2005.
11. Trauer T: **Symptom severity and personal functioning among patients with schizophrenia discharged from long-term hospital care into the community**. *Community mental health journal* 2001, **37**(2):145-155.
12. Trauer T, Farhall J, Newton R, Cheung P: **From long-stay psychiatric hospital to Community Care Unit: evaluation at 1 year**. *Social psychiatry and psychiatric epidemiology* 2001, **36**(8):416-419.
13. Hamden A, Newton R, McCauley-Elsom K, Cross W: **Is deinstitutionalization working in our community?** *International journal of mental health nursing* 2011, **20**(4):274-283.
14. Hobbs C, Newton L, Tennant C, Rosen A, Tribe K: **Deinstitutionalization for long-term mental illness: a 6-year evaluation**. *The Australian and New Zealand journal of psychiatry* 2002, **36**(1):60-66.
15. Farhall J, Trauer T, Newton R, Cheung P: **Minimizing adverse effects on patients of involuntary relocation from long-stay wards to community residences**. *Psychiatric services (Washington, DC)* 2003, **54**(7):1022-1027.
16. Chopra P, Herrman HE: **The long-term outcomes and unmet needs of a cohort of former long-stay patients in Melbourne, Australia**. *Community mental health journal* 2011, **47**(5):531-541.
17. Munro J, Palmada M, Russell A, Taylor P, Heir B, McKay J, Lloyd C: **Queensland extended care services for people with severe mental illness and the role of occupational therapy**. *Australian occupational therapy journal* 2007, **54**:257-265.
18. McKenna B, Oakes J, Fourniotis N, Toomey N, Furness T: **Recovery-Oriented Mental Health Practice in a Community Care Unit: An Exploratory Study**. *Journal of forensic nursing* 2016, **12**(4):167-175.
