# Supplementary material for: A systematic review of service models and evidence relating to the clinically operated community-based residential mental health rehabilitation for adults with severe and persisting mental illness in Australia
Source: BMC Psychiatry. 2019 Feb 4;19:55. doi: 10.1186/s12888-019-2019-5 (PMC6360669; doi:10.1186/s12888-019-2019-5)
Supplement: Supplementary file 5 — Included research relating to community-based and clinically operated residential rehabilitation for people affected by schizophrenia and related disorders in Australia. (PDF 559 kb) [file 12888_2019_2019_MOESM5_ESM.pdf]

Appendix 4: Included research relating to community-based and clinically operated residential rehabilitation for people affected by schizophrenia and related disorders in Australia.

Source^	Objective	Design	Service setting*	Sample characteristics	Brief description>	Quality	Results
Parker et al. (2017 & 2018)[1, 2]	Explore consumers' expectations of community-based recovery-oriented residential rehabilitation units, including comparison with previous mental health care and support.	Mixed methods (qualitative emphasis)	▪ Timeframe: 2014-2016<br>▪ Type: TRR – CCU, ISM<br>▪ Location: Queensland<br>▪ Focus: Severe and Persistent Mental Illness (SPMI)	▪ Consumers entering CCUs operating an ISM (n = 16), and a clinical staffing model (n=8).	▪ Method: Pragmatic approach to grounded theory, semi-structured interviews with consumers that explored how they came to be there and their expectations of the experience. Additionally, statistical comparison was completed on consumers entering the ISM and clinical sites.<br>▪ Comparator(s): Acute / sub-acute inpatient care, supported accommodation, and outpatient care.<br>▪ Outcome(s): N/A.	Good	▪ Characteristics of participants entering the sites were generally comparable. However, ISM participants were significantly less likely to be referred from an inpatient facility and treated with clozapine and had lower levels of medication usage.<br>▪ Consistency emerged across sites, with overarching themes of the expectation of the CCU as a 'transitional place' and a 'transformational space'.<br>▪ All participants expected the CCU to offer an improvement on previous care, including reference to: 'people (staff and co-residents)'; focus of care; 'physical environ'; and 'rules and regulations'.<br>▪ Housing insecurity / homelessness was the most common driver for engagement rather than the opportunity for rehabilitation.<br>▪ Favourable expectations of peer support worker availability under the ISM.
Meurk, Parker, Newman & Dark (nd.)[3]	Examine staff expectations on commencement at community based residential rehabilitation units trialling a novel integrated staffing configuration.	Qualitative	▪ Timeframe: 2014-2015<br>▪ Type: TRR – CCU, ISM<br>▪ Location: Queensland<br>▪ Focus: SPMI	▪ Staff within 6-weeks of commencement (n = 15), including 10 Peer Support Workers and 5 clinical staff.	▪ Pragmatic approach to grounded theory, semi-structured interviews with commencing staff. The interview schedule explored: how the service would compare to work experiences; expectations of the CCU; and why they had chosen to work there.<br>▪ Comparator(s): Acute inpatient care<br>▪ Outcome(s): N/A.	Good	▪ Staff expressed optimism about the potential of the service but also acknowledged uncertainty about how peer and clinical workers would work together and role definition.<br>▪ The CCU was expected to be 'a place of mutual learning and co-development', 'a temporary and transitional place', and to provide a simulacra of community living.
Meehan (2017) et al^[4]	Comparing consumers receiving community-based residential and inpatient rehabilitation care.	Cross-sectional study	▪ Timeframe: 2013<br>▪ Type: TRR – CCU<br>▪ Location: Queensland<br>▪ Focus: SPMI	▪ CCU residents (N = 115)<br>▪ Hospital-based rehabilitation inpatients (N = 125).	▪ Method: Audit of state-wide (Qld) consumers residing at community and hospital-based rehabilitation units, with information sourced from treating staff.<br>▪ Comparator(s): Inpatient rehab.<br>▪ Outcome(s): N/A.	Fair	▪ CCU consumers were significantly younger and less likely to be subject to involuntary treatment or guardianship orders, then people residing in inpatient rehabilitation services.<br>▪ CCU consumers had lower levels of symptoms (HoNOS) and disability (LSP-16) and were less likely to be assessed as being of 'moderate to high risk' of violence than inpatient rehabilitation consumers.
Parker et al. (2016)[5]	Exploring the staff experience of working at a residential rehabilitation service.	Qualitative	▪ Timeframe: 2014-2015<br>▪ Type: TRR – CCU<br>▪ Location: Queensland<br>▪ Focus: SPMI	▪ Nursing, allied health and non-clinical support staff with >12 months experience working in the CCU context (n = 8).	▪ Method: Pragmatic approach to grounded theory, with semi-structured interviews exploring: understanding of the service; comparison to other services; expectations before commencement; and reasons for continuing to work there.<br>▪ Comparator(s): N/A<br>▪ Outcome(s): N/A	Good	▪ Four key themes emerged through the analysis: 'rehabilitation is different to treatment', the CCU as a 'positive transitional space'; 'they (consumers) have to be ready to engage'; and 'recovery is central to rehabilitation practice'.<br>▪ Burnout and external pressures within the mental health system were identified as limiting factors on the ability to maintain recovery-oriented rehabilitation practice.
McKenna et al. (2016)[6]	Exploring at a CCU: what aspects of the current model fit within defined 'recovery' domains; and the 'pragmatic processes' staff use to shape the care they provide.	Qualitative	▪ Timeframe: 2014<br>▪ Type: TRR – CCU<br>▪ Location: Victoria<br>▪ Focus: SPMI	▪ Purposive sampling identifying key stakeholders' (N = 21): consumers (n = 7), informal carers (n = 3) and staff (n = 11).	▪ Method: Thematic analysis, with a general inductive approach, applied in the analysis of one-to-one interviews.<br>▪ Comparator(s): N/A<br>▪ Outcome(s): N/A	Fair	▪ 6 content domains relating to recovery at a CCU were identified: a common vision of recovery as "a continuous journey"; the importance of clinicians 'promoting hope'; shifting emphasis from rehabilitation to 'promoting autonomy and self-determination'; the centrality of meaningful engagement and collaborative partnerships'; 'holistic and personalised care', including family and carer involvement; and 'community participation and citizenship'.<br>▪ Identified clinicians as 'grappling with tension between personal and clinical recovery' concepts, including the role of rehabilitation in preparing some consumers to be 'recovery ready' and 'lack of motivation' as a driver for directive practice.

Chopra et al. (2011)[7]	Assess long-term outcomes and ongoing unmet needs following transfer from long-stay inpatient care to a CCU.	Mixed methods (case series and qualitative)	▪ Timeframe: 1995-2003<br>▪ Type: C-BRC – CCU<br>▪ Location: Victoria<br>▪ Focus: Deinstitutionalisation, psychotic disorders	▪ Initial cohort of consumers transferred from long-stay inpatient care (N = 18),<br>▪ Interviews with consenting cohort members (n=14).	▪ Method: combined retrospective and prospective study, including review of medical records and interviews with surviving cohort members. Analysis at the descriptive level only.<br>▪ Comparator(s): N/A<br>▪ Outcome(s): COLI, HoNOS, LSP-16, WHO LCS	Fair	▪ Eight years following transition to the CCU from long-term inpatient care consumers continue to experience high levels disability, dependence on clinical service, and limited social and family support networks.<br>▪ Themes of disempowerment/dependence, instability of accommodation and social networks, limitations in the consistency of care over time, and loss emerged in the follow-up interviews.
Hamden et al. (2011)[8]	Exploring for current and former CCU residents: quality-of-life; attitudes towards care; and impact on length of stay and re-admission rates.	Pre-post studies with no control	▪ Timeframe: 1996-2007<br>▪ Type: C-BRC - CR<br>▪ Location: Victoria<br>▪ Focus: Deinstitutionalisation, SPMI.	▪ Current (N = 20) and former residents (N = 101) admitted during 1996-2007,<br>▪ Interviews with current (n = 16) and former (n = 15) residents.	▪ Method: Descriptive, exploratory design based on retrospective examination of medical records for demographic data and prospective interviews.<br>▪ Comparator(s): N/A<br>▪ Outcome: PAQ, MANSA	Poor	▪ The CCU environment had a positive impact on friendship and social network building, and reduced LOS and readmissions to acute settings.<br>▪ Positive attitudes of past and current residents towards the CCU were reported.
Barnett et al (2011)[9]	Describing service user characteristics, the nature of service provision, and the implementation experience.	Mixed methods	▪ Timeframe: 2007-2010<br>▪ Type: TRR – CRU<br>▪ Location: South Australia<br>▪ Focus: Schizophrenia and related disorders.	▪ Retrospective administrative data (max n = 238)<br>▪ Survey of staff (n = 43) and key/support workers (n = 132)<br>▪ Consumer (n = 17) and carer (n = 15) focus groups<br>▪ Structured interviews with managers (n =3)	▪ Method: Mixed data derived from retrospective review of administrative records and qualitative data was considered.<br>▪ Comparator(s): N/A<br>▪ Outcome: inpatient bed utilisation, HoNOS	Poor	▪ Reductions in use of inpatient services reported for consumers comparing the 6-months before and after their CRC stay, and in total HoNOS and all associated subscale scores except for behaviour comparing entry and exit.<br>▪ Consumers, carers, staff and community partners generally understood the rehabilitation function of the units.
Smith, Williams & Lefay (2009)[10]	Initial evaluation and description of the implementation experience of a residential rehabilitation service.	Mixed methods	▪ Timeframe: 2006-2008<br>▪ Type: TRR - Hawthorne House<br>▪ Location: Western Australia<br>▪ Focus: Step-down from acute inpatient care for people affected by major mental illness.	▪ Administrative data for admitted clients over the initial 20-months of operation (N = 39)<br>▪ Semi-structured interviews with clients (n = 8), staff (n = 13) and external stakeholders (n=19)<br>▪ Carer survey (n = 4).	▪ Method: Descriptive data of service users is presented alongside qualitative data from interviews and questionnaires with key stakeholders.<br>▪ Comparator(s): N/A<br>▪ Outcome(s): N/A	Poor	▪ No data available to demonstrate objective improvement in functioning or hospital utilisation, however, client feedback reported as 'universally positive'.<br>▪ Carers reflected positively on involvement in and outcomes of care.<br>▪ Staff reported implementation challenges relating to the definition of operational policies and clinical programs, staffing and management.<br>▪ Feedback from community mental health service, non-government agencies and local community organisations was described as positive.<br>▪ Inpatient services reported issues relating to access and the role of the service within the service framework.
Munro et al. (2007)[11]	Describing extended care services (inpatient and community residential), including provision of stakeholder perspectives.	Qualitative	▪ Timeframe: Unspecified<br>▪ Type: TRR – CCU<br>▪ Location: Queensland<br>▪ Focus: SPMI.	▪ Multiple stakeholders: a consumer (n = 1); a carer (n = 1); a 'small interest group' of Occupational Therapists (OTs, undefined).	▪ Method: Narrative perspectives of a single consumer and carer, as well as perspectives of group of Occupational Therapists are presented without methodological description.<br>▪ Comparator(s): Acute inpatient and outpatient care<br>▪ Outcome(s): N/A	Poor	▪ The consumer narrative compared the CCU favourably to acute inpatient care, and emphasised opportunities for activity engagement and independence.<br>▪ The carer perspective valued 'opportunities for stable relationships between clients, staff and families' facilitated by the environment, and having someone else take responsibility for care.<br>▪ OTs described challenges relating to the provision of long term care, including the risk of burnout, and identified two main professional roles: case management and discipline-specific work.
Farhall et al. (2003) [12]	Determine the prevalence of relocation trauma and the impact of the transition process on adjustment.	Prospective Cohort Study	▪ Timeframe: 1994-1999<br>▪ Type: C-BRC – CCU<br>▪ Location: Victoria<br>▪ Focus: Deinstitutionalisation, schizophrenia.	▪ Patients transitioning from long-stay psychiatric wards to CCUs (n = 85˜).	▪ Method: Pre-post comparison of multiple assessments completed one month before and after transition.<br>▪ Comparator(s): Transitional ward and Long-stay inpatient care (RPP data only).<br>▪ Outcome(s): PANSS, SOAS, PAQ, RPP	Good	▪ Most patients preferred the CCU to previous hospital-based care (66%). CCU based care was significantly less restrictive than that in the transitional wards and long stay units.<br>▪ Relocation trauma affected 25% of patients.<br>▪ Variables associated with reduced likelihood of relocation trauma were: preparation in a transition unit, making 6 or more pre-move visits, and having a preparation period greater than 16 weeks.

Hobbs et al. (2002)[13]	6-year follow-up of the consumers transferred from long-term inpatient care to community residences, considering: ongoing accommodation and care needs; clinical change; and service user perspectives.	Mixed methods (Prospective cohort and Qualitative)	▪ Timeframe: 1994-2000<br>▪ Type: C-BRC – CR<br>▪ Location: New South Wales<br>▪ Focus: Deinstitutionalisation, schizophrenia.	▪ Formerly institutionalised consumers transferred to a community residence (n = 47).	▪ Method: Repeat measures analysis was applied to evaluate change in outcomes over time. Also, semi-structured interviews were used to explore service-user perspectives.<br>▪ Comparator(s): N/A<br>▪ Outcome(s): BPRS, CPZ Eq., LSP, MADRAS, QOL, SBS	Fair	▪ Most residents were still in the community 6-years following transition (n = 40/47, 85%)<br>▪ Community residents report decreasing levels of activity engagement over-time, with them 'valuing the greater freedom of independence' but also describing difficulties in 'enhanc[ing] their own social networks'.<br>▪ 18% required re-admission within 2-years of transition, and 28% required readmission in the subsequent 4-years. Pre-transition BPRS scores were significantly higher for participants in hospital care at the 6-year follow-up.<br>▪ No significant differences between hospital and community-based residents were present at 6-year follow-up. No community-based residents required ongoing 24-hour supervision, 64% required daily case management support and 36% lived semi-independently needing only weekly or monthly case-management support.<br>▪ Significant improvements in overall quality of life and reductions in CPZ eq were noted for community-based residents over the 6-year follow-up.
Trauer et al. (2001)[14]	Explore symptoms and functioning in consumers transferred from long-term inpatient care to a CCU.	Case Series	▪ Timeframe: 1997-1998<br>▪ Type: C-BRC – CCU<br>▪ Location: Victoria<br>▪ Focus: Deinstitutionalisation, schizophrenia.	▪ Cohort of established residents (n = 20) recently discharged from long-stay inpatient care.	▪ Method: Repeated assessment of symptoms and functioning 1-year apart.<br>▪ Comparator(s): N/A<br>▪ Outcome(s): MCAS, PANSS	Good	▪ No significant differences in symptoms and functioning were observed after one year of CCU based care.
Trauer, Farhall, Newton & Cheung (2001)[15]	Examine the transition from long-stay institutional care to community residence over a one-year period.	Pre-post study with no control	▪ Timeframe: unspecified<br>▪ Type: C-BRC - CCU<br>▪ Location: Victoria<br>▪ Focus: Deinstitutionalisation, schizophrenia.	▪ All patients residing on long-stay institutional wards proposed for transfer to CCUs (n = 125).	▪ Method: Repeated assessment of symptoms, functioning and care environment pre-move and 1-year post.<br>▪ Comparator(s): N/A<br>▪ Outcome(s): LSP, LQoLP, PANSS, PAQ, RPQ, SOAS, SNA	Poor	▪ Residents, relatives and carers preferred Community-based care, and these settings were viewed as less regimented than ward-based care.<br>▪ No change in symptoms and functioning, but with improvements noted in quality of life.
Newton et al. (2000)[16]	Describe the experience of participants transitioning from long-term inpatient care to community residence.	Qualitative	▪ Timeframe: 1994-1996<br>▪ As per Hobbs et al (2002)	▪ Observations, interactions and information relating to multiple stakeholders (residents, staff, peers and community members).	▪ Method: Ethnographic approach included participant observational fieldwork, open-ended and semi-structured interviews life history taking, and perusal of written records.<br>▪ Comparator(s): N/A<br>▪ Outcome(s): N/A	Poor	▪ Residents report a preference for community living over long-term inpatient care.<br>▪ Acquiring new skills is not always easy for residents transitioning from long-term institutional care.<br>▪ Over time, staff acknowledged slow but continual progress and decreasing support needs, and consumers were increasingly accepted by peers and community members.

Notes

^ Only research components presenting original data are included (i.e. systematic and narrative review data, opinions etc. are excluded)

* See also Figure 2 and Appendix 2 for further detail, C-BRC - Community-Based Residential Care; CCU – Community Care Unit; CR- Community Residences; CRC- Community Rehabilitation Centre; ISM – Integrated Staffing Model, SPMI – Severe and Persistent Mental Illness, TRR - Transitional Residential Rehabilitation

Additional available information about consumer characteristics is presented in Table 3, including additional data from the same benchmarking series described by Meehan et al. (2017)

~ Note that while 87 patients transitioned 2 were excluded from the cohort (1x re-admission, 1x death). Also, data on relocation trauma was only available for a subset of consumers (n = 81).

> COLI, CPZ Eq. - ChlorPromaZine dose Equivalence, HoNOS - Health of the Nation Outcome Scale, LQoLP - Lancashire Quality of Life Profile, LSP - Life Skills Profile, LSP-16 - Life Skills Profile 16, MADRAS - Montgomery-Asberg Depression Rating Scale, MANSA - Manchester Short Assessment for Quality of Life, MCAS - Multnomah Community Ability Scale, PANSS - Positive and Negative Symptoms Scale, PAQ - Patient Attitude Questionnaire, QOL - Quality Of Life index, RPQ - Rehabilitation Practices Questionnaire, RPP - Residential Practices Profile, SBS - Social Behaviour Scale, SNA - Social Network Assessment, SOAS - Staff Observation Aggression Scale, WHO LCS - WHO Life chart schedule.

REFERENCES

1. Parker S, Meurk C, Newman E, Fletcher C, Swinson I, Dark F: **Understanding consumers' initial expectations of community-based residential mental health rehabilitation in the context of past experiences of care: A mixed-methods pragmatic grounded theory analysis**. *International journal of mental health nursing* 2018.
2. Parker S, Dark F, Newman E, Hanley D, McKinlay W, Meurk C: **Consumers' understanding and expectations of a community-based recovery-oriented mental health rehabilitation unit: a pragmatic grounded theory analysis**. *Epidemiology and psychiatric sciences* 2017:1-10.
3. Meurk C, Parker S, Newman E, Dark F: **Staff expectations of an integrated model of residential rehabilitation for people with severe and persisting mental illness: A pragmatic grounded theory analysis**. *Health & Social Care in the Community* nd., **Under review**.
4. Meehan T, Stedman T, Parker S, Curtis B, Jones D: **Comparing clinical and demographic characteristics of people with mental illness in hospital- and community-based residential rehabilitation units in Queensland**. *Aust Health Rev* 2017, **41**(2):139-143.
5. Parker S, Dark F, Newman E, Korman N, Rasmussen Z, Meurk C: **Reality of working in a community-based, recovery-oriented mental health rehabilitation unit: A pragmatic grounded theory analysis**. *International journal of mental health nursing* 2017, **26**(4):355-365.
6. McKenna B, Oakes J, Fourniotis N, Toomey N, Furness T: **Recovery-Oriented Mental Health Practice in a Community Care Unit: An Exploratory Study**. *Journal of forensic nursing* 2016, **12**(4):167-175.
7. Chopra P, Herrman HE: **The long-term outcomes and unmet needs of a cohort of former long-stay patients in Melbourne, Australia**. *Community mental health journal* 2011, **47**(5):531-541.
8. Hamden A, Newton R, McCauley-Elsom K, Cross W: **Is deinstitutionalization working in our community?** *International journal of mental health nursing* 2011, **20**(4):274-283.
9. Barnett K, Guiver N, Cheok F: **Evaluation of the Three Community Rehabilitation Centres: FINAL REPORT**. In. Edited by Health S. South Australia: SA Health; 2011.
10. Smith G, Williams T, Lefay L: **Evaluating the Hawthorn House Rehabilitation Service**. In. Perth: Department of Health; 2009.
11. Munro J, Palmada M, Russell A, Taylor P, Heir B, McKay J, Lloyd C: **Queensland extended care services for people with severe mental illness and the role of occupational therapy**. *Australian occupational therapy journal* 2007, **54**:257-265.
12. Farhall J, Trauer T, Newton R, Cheung P: **Minimizing adverse effects on patients of involuntary relocation from long-stay wards to community residences**. *Psychiatric services (Washington, DC)* 2003, **54**(7):1022-1027.
13. Hobbs C, Newton L, Tennant C, Rosen A, Tribe K: **Deinstitutionalization for long-term mental illness: a 6-year evaluation**. *The Australian and New Zealand journal of psychiatry* 2002, **36**(1):60-66.
14. Trauer T: **Symptom severity and personal functioning among patients with schizophrenia discharged from long-term hospital care into the community**. *Community mental health journal* 2001, **37**(2):145-155.
15. Trauer T, Farhall J, Newton R, Cheung P: **From long-stay psychiatric hospital to Community Care Unit: evaluation at 1 year**. *Social psychiatry and psychiatric epidemiology* 2001, **36**(8):416-419.
16. Newton L, Rosen A, Tennant C, Hobbs C, Lapsley HM, Tribe K: **Deinstitutionalisation for long-term mental illness: an ethnographic study**. *The Australian and New Zealand journal of psychiatry* 2000, **34**(3):484-490.
